# Comparative MS- and NMR-Based Metabolome Mapping of Egyptian Red and White Squill Bulbs F. Liliaceae and in Relation to Their Cytotoxic Effect

**DOI:** 10.3390/plants12112078

**Published:** 2023-05-23

**Authors:** Omar M. Khattab, Dina M. El-Kersh, Shaden A. M. Khalifa, Nermeen Yosri, Hesham R. El-Seedi, Mohamed A. Farag

**Affiliations:** 1 Department of Chemistry, Faculty of Science, Menoufia University, Shebin El-Kom 32512, Egypt; omarkhattab500@gmail.com; 2 Department of Pharmacognosy, Faculty of Pharmacy, The British University in Egypt, Cairo 11837, Egypt; dina.elkersh@bue.edu.eg; 3 Psychiatry and Psychology Department, Capio Saint Göran’s Hospital, Sankt Göransplan 1, 112 19 Stockholm, Sweden; shaden.khalifa@regionstockholm.se; 4 Chemistry Department of Medicinal and Aromatic Plants, Research Institute of Medicinal and Aromatic Plants (RIMAP), Beni-Suef University, Beni-Suef 62514, Egypt; nermeen.yosri@rimp.bsu.edu.eg; 5 International Research Center for Food Nutrition and Safety, Jiangsu University, Zhenjiang 212013, China; 6 International Joint Research Laboratory of Intelligent Agriculture and Agri-Products Processing, Jiangsu Education Department, Zhenjiang 212013, China; 7 Pharmacognosy Department, College of Pharmacy, Cairo University, Kasr El Aini St., Cairo 11562, Egypt

**Keywords:** *Urginea martima*, squill, bufadienolides, SPME-GC/MS, NMR, UPLC/MS, metabolomics

## Abstract

*Urginea maritima* L. (squill) species is widely spread at the Mediterranean region as two main varieties, i.e., white squill (WS) and red squill (RS), that are recognized for several health potentials. The major secondary metabolite classes of the squill are cardiac glycosides, mainly, bufadienolides, flavonoids, and anthocyanins. Herein, a multiplex MS and NMR metabolomics approach targeting secondary and aroma compounds in WS and RS was employed for varieties classification. Solid-phase micro extraction-gas chromatography/mass spectroscopy (SPME-GC/MS), ultra-high-performance liquid chromatography/mass spectrometry (UPLC/MS), as well as nuclear magnetic resonance (NMR) provided fingerprinting and structural confirmation of the major metabolites for both types of the squill. For comparison of the different platforms’ classification potential, multivariate data analysis was employed. While Bufadienolides, viz. “hydroxy-scilliglaucosidin-*O*-rhamnoside, desacetylscillirosidin-*O*-rhamnoside and bufotalidin-*O*-hexoside” as well as oxylipids, were enriched in WS, flavonoids, i.e., dihydro-kaempferol-*O*-hexoside and its aglycon, taxifolin derivative, were predominant in RS. A cytotoxicity screening against three cancer cell lines, including breast adenocarcinoma (MCF-7), lung (A-549), and ovarian (SKOV-3) cell lines was conducted. Results revealed that WS was more effective on A-549 and SKOV-3 cell lines (WS IC_50_ 0.11 and 0.4 µg/mL, respectively) owing to its abundance of bufadienolides, while RS recorded IC_50_ (MCF7 cell line) 0.17 µg/mL since is is rich inflavonoids.

## 1. Introduction

*Urginea*, closely related to the “Drimia” genera belonging to family Asparagaceaeis, is widely spread at the Mediterranean region, Africa and India [1]. In Arabic, it is known as Basal farion, Onsul, and Samm el-Far [2]. Other common names are termed squill and sea onion [3]. *U. martima* species comprises two varieties to include white and red, with both recognized for their bulb’s medicinal value and abundance of cardiac glycosides [4,5]. Stoll and coworkers isolated cardiac glycosides of *U. maritima* for the first time in structure similar to that of the Bufo genus termed bufadienolides, though it was less potent than digitalis cardiac glycosides [6]. *U. maritima* bulbs displayed a wide spectrum of pharmaceutical potentials. *U. maritima* bulbs enriched with “cardiac glycosides” Bufadienolides, *viz.* glucoscillarene A, proscillaridine A, scillarene A, scilliglaucoside and scilliphaeoside. Those bufadienolides involved in the cardiovascular system (CVS) via inhibiting Na^+^/K^+^ ATPase, which increases intracellular Ca^+2^, leading to enhancement of the contractility of cardiac muscles (positive inotropic effect) [6,7]. The methanolic extract and methylene chloride fraction of *Urginea* bulbs were reported as being antibacterial, anthelmintic, diuretic, and expectorant. Fresh bulbs were used for wound healing [7,8]. Besides its CVS effect, it possesses potential cytotoxic activity against lymphoma and breast cancer human cell lines in addition to its antirheumatic and antimicrobial actions [1,3,7]. The red variety of *U. maritima* differs from white squill mostly by the presence of red pigments, i.e., anthocyanins. Anthocyanins, such as cyanidin-3-monoglucoside and pelargonidin-3-monoglucoside, either free or acylated with caffeic or *p*-coumaric acid, were previously reported from the red bulbs of Spanish *U. maritima*. Taxifolin, taxifolin-4’-glucoside, quercetin-3-glucoside, C-flavone glycosides, and caffeic acid were isolated as major phenolic components [9]. Traces of fixed oil and volatile oil were also reported in addition to lignans and fatty acids [1,2,3,7,10,11,12]. Egyptian *U. maritima* showed more complex types of bufadienolides from its bulbs, which are from other *Urginea* species collected from Turkey, Greece and Tunisia [13]. Herein, we aimed to adopt a metabolomics approach for the classification of RS and WS, targeting their metabolome, including volatiles, as well as the secondary metabolites via multiple analytical platforms, viz. SPME/GC-MS, 1D, 2D NMR, and UPLC-MS, respectively. For data visualization and untargeted classification, multivariate data analyses, unsupervised learning, i.e., principal component analysis (PCA), and supervised learning, i.e., orthogonal projection to latent structures discriminant analysis (OPLS-DA) models, were applied. Both PCA and OPLS focus on identifying variances and similarities among specimens as extensively reported in our similar previous work using the same comparative MS and NMR platforms in metabolomics analysis of rosella [14,15,16].

## 2. Results and Discussion

### 2.1. Identification of Volatile Organic Compounds (VOCs)

**Figure 1:**
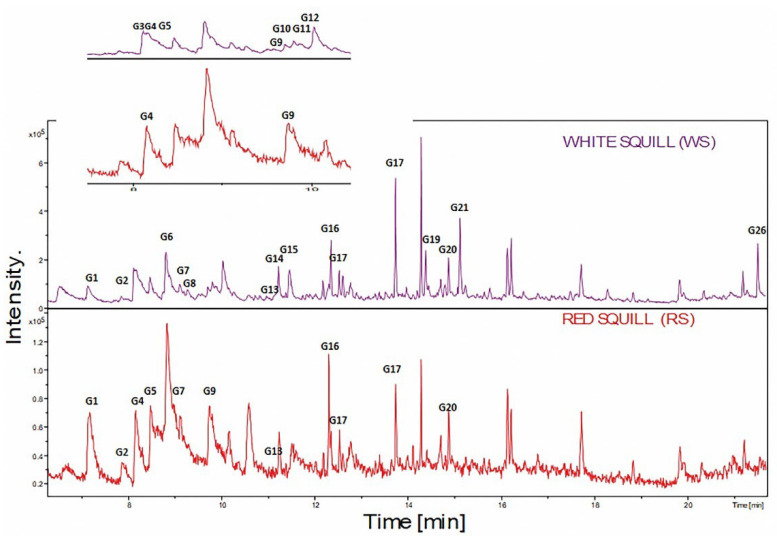
SPME-GC/MS chromatogram of WS and RS headspace volatiles. The corresponding volatile names for each peak followed that listed in Table 1.

The volatiles profiling of RS and WS was determined using headspace SPME coupled with GC-MS, as reported in the current study, for the first time. A total of 26 volatiles were identified belonging to monoterpenes (10), aliphatic hydrocarbons (6), oxygenated compounds (4), in addition to a heterocyclic, an aromatic, and a sesquiterpene, as listed in Table 1. Due to the higher percentiles of monoterpenes and aliphatic hydrocarbons in RS compared to WS (47.82% and 37.26% vs. 25.69% and 19.76%), respectively, RS had a stronger aroma profile than WS. Other classes found exclusively in WS included heterocyclics, oxygenated hydrocarbons, aromatics, sesquiterpenes, oxygenated monoterpenes, ca. 4.57%, 12.15%, 1.75%, 8.22% and 2.29%, respectively, as shown in Figure 1.

### 2.2. Secondary Metabolites Profiling via UHPLC/MS

**Figure 2:**
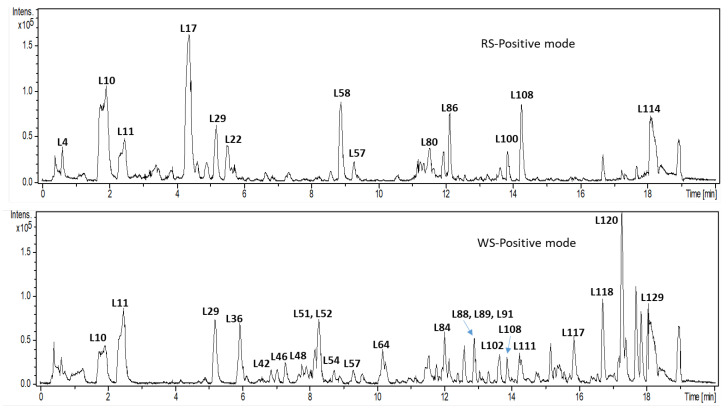
UPLC/MS base peak chromatogram of both RS and WS squill varieties. Detections in “positive ionization mode” showing major secondary metabolites.

UPLC-MS profiling was employed for secondary metabolites, and profiling in WS is denoted in pink color versus RS in red color. Figure 2 highlights the major differences between the two varieties. Diversity of secondary metabolite classes were identified in both squill, including mostly cardiac glycosides “bufadienolides” (50) followed by flavonoids (40) including“flavonols, flavanones and dihydroflavonols”. Minor classes of coumarins (1), anthocyanins (2), and phenolic acids (5) were detected, with full spectral data of identified peaks presented in Table 2.

The UV spectra of the major classes of secondary metabolites were observed at *λ* 280–299 nm. For bufadienolides glycosides, they were observed at 292–299 nm [17], while phenolics showed UV max at 280–283 and 296–340 nm [18]. Different classes have been annotated in both white and red squill, *viz.* bufadienolides, flavonoids, phenolic, amino, and fatty acids, as detailed in the next subsections. All identified secondary metabolites were listed in Table 2 with Supplementary Figures showing typical fragmentation pattern supporting their identification.

#### 2.2.1. Bufadienolides

**Figure 3:**
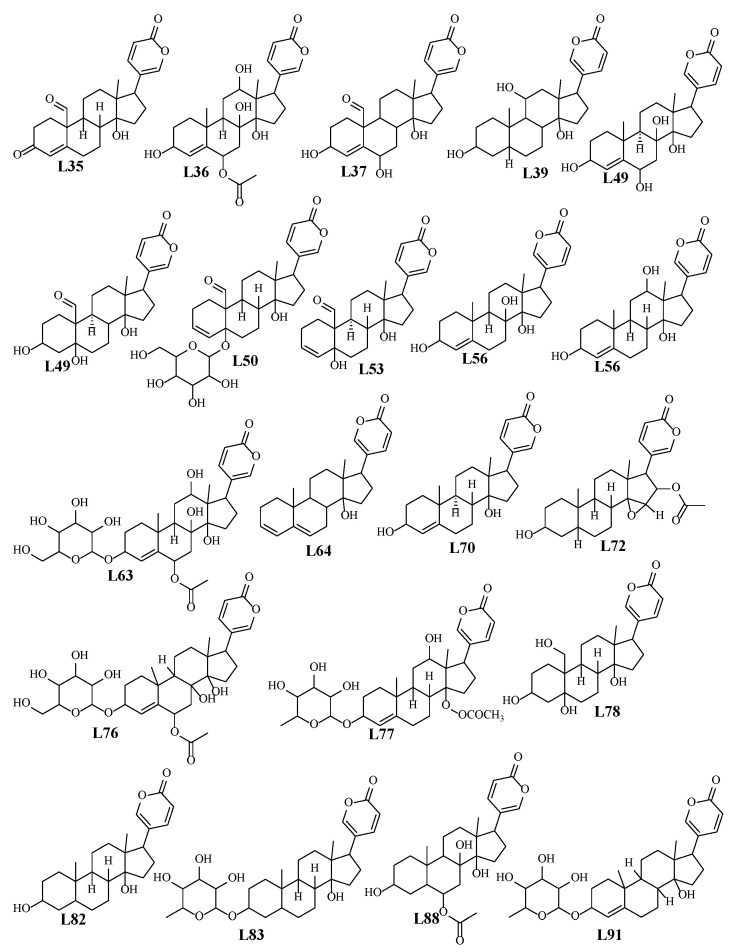
Chemical structure of bufadienolides in both RS and WS detected using UPLC/MS.

Bufadienolides are C-24 steroids with a pyranone ring at C-17*β* that are naturally present in plants and mammalian animals, specifically toads and snakes [19]. Bufadienolides were found more abundant in WS than RS as revealed from the inspection of the chromatograms of both WS and RS at retention time “R_t_.” from 5 to 13 min. A series of different classes of bufadienolides were identified in WS and RS and included hydroxy-oxobufadienolide with a C-19 aldehyde group, i.e., bufotalidin, and hydroxybufaenolides with a C-19 methyl group, including scillarenin and scilliphosidin. Notably, identified bufadienolides structures were illustrated in Figure 3.

##### Hydroxyoxobufaenolide

Hydroxyoxobufaenolides were detected in multiple peaks L53, L37, L49, while L36, L63, L67 and L68 were annotated as glycosides of hydroxyoxobufaenolides [20,21,22]. L36, L63, L67, and L68 were identified as hydroxyoxobufaenolide glycosides based on the loss of −162 or 146 amu. in L36 desacetylscillirosidin-*O*-thevetoside (*m/z* 577, (C_31_H_44_O_10_)^+^) (Figure S1A), L63 Hydroxyscilliroside (*m/z* 637, (C_32_H_44_O_13_)^+^) (Figure S1B) [23], L67 m/z 579 bufatalidin-*O*-hexoside, and L68 *m/z* 545 (C_30_H_41_O_9_)⁺ [M+H]^+^ scilliglaucosidin-*O*-rhamnoside (Figure S1C).

Identified hydroxybufaenolides are subdivided by the number of hydroxyl groups to mono, di, tri and tetra hydroxyl derivatives and were identified in both WS and RS as listed in Table 2.

The L64 peak *m/z* 367 (C_24_H_31_O_3_)^+^ [M+H]^+^ was detected in both WS and RS as monohydroxybufa-tetraenolide “Scillaridin A” [24,25,26] as an example of a monohydroxybufa-enolide, illustrated in Figure S1D.

Dihydroxybufaenolides were detected in several peaks including L82 *m/z* 387 (C_24_H_35_O_4_)^+^ [M+H]^+^ dihydroxybufadienolide “bufalin” [27] and L70 *m/z* 385 (C_24_H_33_O_4_)^+^ [M+H]^+^ dihydroxy-bufa-trienolide “Scillarenin” [28,29,30]. Glycosidic conjugates were observed in L91 *m/z* 531, showing a loss of rhamnose (−146 amu) (Figure S1E) and L86 *m/z* 693 (C_36_H_53_O_13_)^+^ [M+H]^+^ with a loss of hexose (−162 amu) (Figure S1F). L91 and L86 annotated as scillarenin-*O*-rhamnoside “Proscillaridin A” [11,31,32,33] and proscillaridin A-*O*-glucoside, respectively.

Acylated bufaenolides were detected in L90 at *m/z* 607 (C_32_H_47_O_11_)⁺ [M+H]^+^ and annotated as acetyl-scilliphaeoside. L39 at *m/z* 403 (C_24_H_35_O_5_)^+^ [M+H]^+^ annotated as trihydroxybufa-dienolide “gamabufotalin” [29,34,35] (Figure S1G).

Other acetylated tetrahydroxybufaenolide were detected in peak L54 at *m/z* 477, (C_26_H_37_O_8_)^+^ (M+2H)^+^ identified as hydroxyscillirosidin with a loss of acetyl group likely at *C*-16 [26,36,37] (Figure S1H).

#### 2.2.2. Flavonoids

Compared to WS’s abundance of bufadienolides, flavonoids were more abundant in RS, with several being reported for the first time in this current study. Beside a positive mode, improved detection of flavonoids was observed in a negative mode, with MS2 fragments found to be characteristic of flavonoids, i.e., amu 151, 125 and 153 as well as neutral loss of amu −28 “CO”, −18 “H_2_O” and −44 “CO_2_” [38,39]. The next subsection shall summarize identification of the different flavonoid subclasses and distribution in *Urginea* species.

##### Identification of Flavanols/Flavonols

Identified flavanols in L22 and its methylated derivative in L24 at *m/z* 305 (C_15_H_13_O_7_) ^+^ [M+H] ^+^ and *m/z* 319 (C_16_H_15_O_7_)^+^ were annotated as dihydroquercetin (taxifolin) and dihydroisorhmentin (methyl taxifolin), respectively [40]. Flavonols were also detected in peak L40 at *m/z* 303 (C_15_H_11_O_7_)^+^ [M+H]^+^ identified as quercetin, as well as its glycosides L41 and L32 (Table 2).

Another major flavanol was detected in peak in RS L17 *m/z* 289 (C_15_H_13_O_6_)^+^ [M+H]^+^ and N13 *m/z* 287 (C_15_H_11_O_6_)^−^ [M-H]^−^ and were annotated as dihydrokaempferol [29,41,42] along with its glycoside in L16 (Figure S1I), N6 (Figure S1J), N7, N9 and N10. Furthermore, catechin and its hexoside belonging to flavanols, (C_21_H_23_O_11_)^−^ and (C_15_H_15_O_6_)^+,^ were detected in red squill as N3 and L14 (*m/z* 451 and 291), respectively [43].

Flavonols, such as N16 *m/z* 465 (C_21_H_21_O_12_)^−^ and N24 *m/z* 447 (C_21_H_19_O_11_)^−^ with loss of 180 amu (hexose + H_2_O) moieties and −162 hexose moiety, were identified as kaempferol-*O*-glucoside [44] (Table 2).

##### Identification of Flavones/Flavanones

Compared to the abundance of flavonols, the flavone subclass was detected in few peaks exemplified in L20 at m/z 271 (C_15_H_11_O_5_)^+^ [M+H]^+^ apigenin and its sugar glycoside in L28 and L33 (Table 2) [41]. L93 m/z 285 (C_16_H_13_O_5_)^+^ was interpreted as dihydroxymethoxyisoflavone [45].

L94 m/z 317 (C_17_H_17_O_6_)^+^, annotated as dihydroxy dimethoxy flavanone, represented an example of flavanone as well as N22 *m/z* 595 (C_27_H_31_O_15_)^−^, with MS_2_ *m*/*z* 271 attributed to the loss of two hexose units (−324 amu) and annotated as naringenin-*O*-dihexoside [46].

Finally, N12 *m/z* 479 (C_22_H_23_O_12_)^−^ is annotated as noidesol A or B [47], and this is the first report of Noidesol in *Urginea* species (Figure S1K).

Compared to flavonols richness in red squill, anthocyanins were likewise identified exclusively in RS, accounting for its characteristic reddish color. Major anthocyanins included L3 at *m/z* 475 (C_21_H_31_O_12_)^+^, annotated as hydroxycinnamyl-*O*-dihexoside [9] (Figure S1L), and L13 at *m/z* 307 (C_15_H_15_O_7_)^+^ [M+H]^+^, identified as leucocyanidin [3] (Figure S1M).

##### Identification of Coumarins

The hydroxycoumarin class has been annotated in both squill types, represented as L29 and N15 *m/z* 163 (C_9_H_7_O_3_)^+^ and 161 (C_9_H_5_O_3_)^−^, respectively, and is in agreement with previous reports for coumarins in *U. indica* species [48,49].

#### 2.2.3. Phenolic Acids

Phenolic acids are aromatic secondary plant metabolites found ubiquitously in plants and play a role in food quality and their organoleptic properties [50]. The primary detected phenolic acid was vanillic acid in L26 at *m/z* 169 (C_8_H_9_O_4_)^+^, and several glycosidic conjugates in peaks L25, L27, and N5, showing *MS_2_* ion fragments of vanillic acid. L25 at *m/z* 447 (C_20_H_29_O_13_)^+^ vanillic-*O*-rhamnosyl-*O*-hexoside, L27 *m/z* 331 (C_14_H_19_O_9_)^+^ vanillic acid-*O*-hexoside [51], and N5 at *m/z* 491 (C_20_H_27_O_14_)^−^ as vanillic acid-*O*-dihexoside with *MS_2_ m/z* 167 [26] in a negative ionization mode was detected. Other detected phenolic acids included in L105 at *m/z* 329 (C_17_H_29_O_6_)^+^ as spiculisporic acid and N14 at *m/z* 473 (C_21_H_29_O_12_)^−^, a glycoside of dimethoxyhydrocinnamic acid [52].

#### 2.2.4. Amino Acids and Fatty Acids

Few amino acids were identified in both squills, including inL12 at *m/z* 205 (C_11_H_13_N_2_O_2_)⁺, L8 at *m/z* 182 (C_9_H_12_NO_3_)^+^, and L9 *m/z* 166 (C_9_H_12_NO_2_)^+^ for tryptophan, tyrosine, and phenyl alanine, respectively. Compared to amino acids showing earlier elution, fatty acids were observed at the end of the chromatogram, considering their lypophilic nature in both varieties. An example of major fatty acids includes L114 at *m/z* 279 (C_18_H_31_O_2_)^+^ [M+H]^+^ octadecatrienoic acid and L120 at *m/z* 281 (C_18_H_33_O_2_)^+^ linoleic acid. No difference in amino acids was observed among squill varieties compared to flavonoids; however, they later appear as stronger marker for variety type.

### 2.3. Multivariate Data Analysis of UPLC-MS Dataset

**Figure 4:**
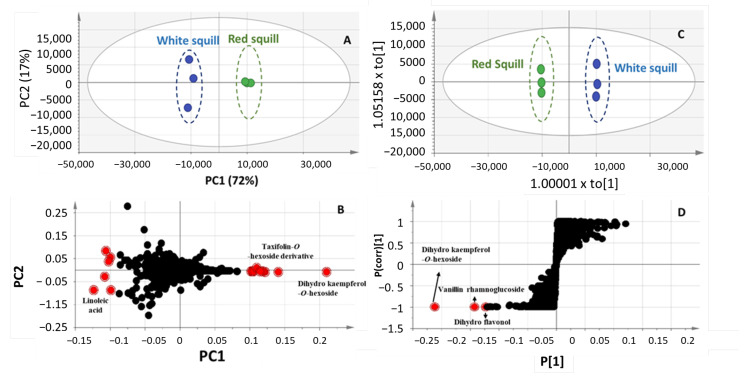
PCA scoring plot (**A**), loading plot, (**B**) OPLS-DA scoring (**C**), and S-plot (**D**) models in negative ionization mode of RS and WS squill varieties.

**Figure 5:**
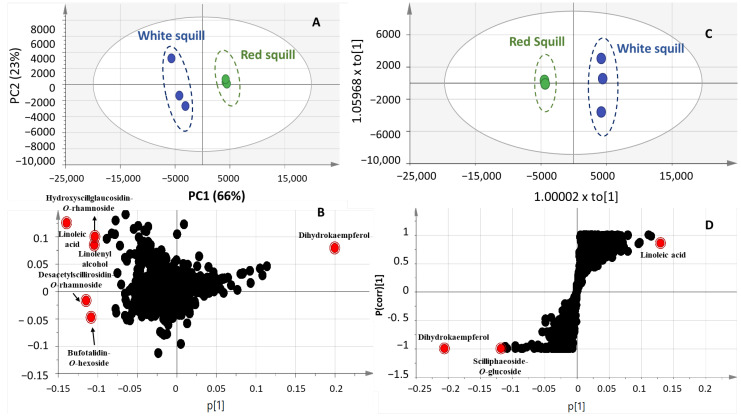
PCA scoring plot (**A**), loading plot (**B**), OPLS-DA Scoring (**C**), and S-plot (**D**) models in positive ionization mode of RS and WS squill varieties.

To aid in identifying further markers for each squill variety in an untargeted manner, unsupervised principle component analysis (PCA) and orthogonal projection to latent structures analysis (OPLS-DA) were attempted in both negative (Figure 4) and positive ionization modes (Figure 5). Complete segregation between the two squill varieties was observed, highlighting that RS was more rich in phenolics, whereas WS was abundant in bufadienolides.

A score plot model derived from the negative ionization mode of UHPLC/MS prescribed by PC1 and PC2 accounted for 72 and 17%, respectively, of the total variance (Figure 4A), with clear segregation of RS from WS. As for revealing the metabolites mediating RS and WS segregation, a PCA-loading plot (Figure 4B) revealed an abundance of flavanols, identified as dihydrokaempferol-*O*-hexoside and taxifolin-*O*-hexoside derivatives, in RS, which are likely to serve as precursors for anthocyanins solely found in RS variety. The OPLS-DA model (Figure 4C) further confirmed the PCA results from the *S*-plot (Figure 4D) revealing that dihydrokaempferol-*O*-hexoside, its aglycon, and vanillin rhamnoglucoside, were abundant in RS compared to WS.

The results from the UHPLC/MS-derived model in the positive ionisation mode were comparable to those in the negative mode (Figure 5A). The two squill varieties, RS and WS, were clearly separated in the PCA model, which was prescribed by PC1 66% and PC2 23%. Furthermore, according to Figure 5B, in accordance with the negative ionisation mode, RS was more abundant in dihydrokaempferol, which is a biosynthetic precursor for anthocyanins in RS [53]. On the other hand, WS had higher concentrations of bufadienolides, such as hydroxy-scilliglaucosidin-*O*-rhamnoside, desacetylscilliglaucosidin-*O*-rhamnoside, and bufotalidin-*O*-hexoside as well as oxylipids, such as linoleic acid and linoleyl alcohol. Supervised OPLS-DA model. Figure 5C,D confirmed linoleic acid enrichment in WS and identifying dihydro-kaempferol “aromandrin” and the bufadienolide “scillipheoside-*O*-glucoside” as markers for RS.

### 2.4. NMR Metabolites Fingerprinting

**Figure 6:**
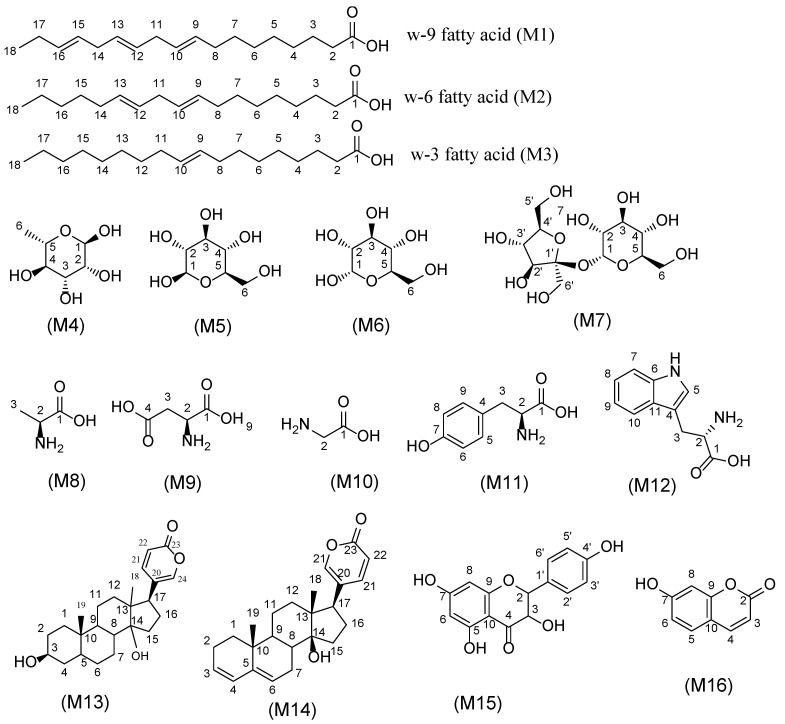
Structure of the major primary and secondary metabolites detected in squill. Metabolite numbers follow those listed in Table 3 for metabolite identification using 1D and 2DNMR.

**Figure 7:**
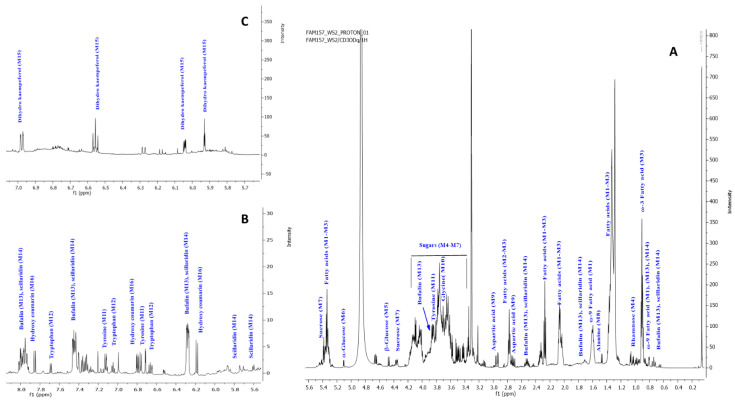
^1^H-NMR of WS extract. (**A**) WS at δ 0–5.6 ppm (**B**) at δ 5.6–8.00 ppm. (**C**) RS at δ 5.7–7.0 ppm, prescribing characteristic signals for primary and secondary metabolites. Peaks annotated at the spectra labeled as follows: ω-9 fatty acid (M1), ω-6 fatty acid (M2), ω-3 fatty acid (M3), rhamnose (M4), *β*-glucose (M5), *α*-glucose (M6), sucrose (M7), alanine (M8), aspartic acid (M9), glycine (M10), tyrosine (M11), tryptophan (M12), bufalin (M13), scillaridin (M14), dihydro kaempferol (M15), and hydroxy coumarin (M16). The compounds spectral data were listed in Table 3.

To provide a broader coverage of squill metabolome, NMR was employed to provide insight on both secondary and primary metabolites, especially with the later class not detected using LCMS. NMR offers also improved structural elucidation tool aided by its extensive 2D NMR experiments and quantitative determination of the major metabolites [54] for quality control purposes. Major classes detected in squill using NMR included sugars, flavonoids, bufadienolides, phenolics, and amino and fatty acids (Figure 6 and Table 3). Nevertheless, compared to MS, NMR suffers from low sensitivity and from signal overlap, especially in aliphatic regions. To overcome the problem of signal overlap, 2D NMR experiments were employed to allow for the resolution of overlapped signal along the second dimension, i.e., carbon in the case of HMBC [55,56]. ^1^H NMR spectra from WS showed the signals relative richness in both varieties (Figure 7 and Table S1).

#### 2.4.1. Fatty Acids

A key feature of the assignment of unsaturated fatty acids (M1, M2 and M3) was based on the signals (-CH_3_) δH 0.89 ppm, a long chain of methylene groups (-CH_2_)_n_ at δH 1.2 ppm, and olefinic bond (s) at δH 5.30–5.37 ppm, and HSQC cross-peak correlation with ^13^C showed signals at δ 15.7, 31.9, 128.8–132.1 ppm, respectively. Additionally, confirmation of unsaturation in fatty acids was based on allylic methylenes resonating at δ 2.05–2.10 ppm and correlated with ^13^C at δ 29.4 ppm. Bis allylic (-CH_2_) with two triplets at δ 2.76 and 2.78 with ^13^C at δ 26.2 and 27.8 ppm were correlated to ω-6 (M2) and ω-3 (M3) fatty acids, respectively. Furthermore, confirmation of these data was done using other 2D NMR experiments, as HSQC and HMBC displayed in Figures S2–S4.

#### 2.4.2. Sugars

Rhamnose (M4), *β*-glucose (M5), *α*-glucose (M6), and sucrose (M7) were the major sugars detected in squill using NMR. Rhamnose (M4) was assigned based on its terminal CH_3_ at δH 1.22 and ^13^C at δ 13.8 ppm showing COSY correlation to anomeric proton at δ 3.34 and 13.51 ppm. Moreover, M5, M6, and M7 were identified based on the anomeric protons at δ 4.48 (d, *J* = 7.8 Hz), 5.10 (d, *J* = 3.7 Hz), and 5.39 (d, *J* = 3.8 Hz) ppm, respectively, alongside their respective ^13^C at δ 99.5, 95.2, and 94.8 ppm detected in HSQC (Figure S5).

#### 2.4.3. Amino Acids

A total of five amino acids were identified in squill, including alanine (M8), aspartic acid (M9), glycine (M10), tyrosine (M11), tryptophan (M12). In details, alanine (M8) showed methyl group signals at *δ* 1.47 (d, *J* = 7.2 Hz) ppm. Moreover, M9, M10, and M11 showed methylene group signals at δ 2.95, 3.87, and 2.79 ppm. In Figure S2, the previously mentioned protons signals showed HSQC cross-peak correlation with ^13^C at δ 36.8, 45.1, and 37.3 ppm for M9 and M10, respectively. There were long-range HMBC correlations with carbons at δ 51.47 for alanine (M8) (Figure S3) while 52.6, 174.9, and 174.5 for aspartic acid (M9) (Figures S3 and S4) and 53.5 for tyrosine (M11) confirmed their assignments. Furthermore, a 1,4 di-substitution benzene ring appearing at δH 6.70 and 7.12 (d, *J* = 8.6 Hz) showed that HSQC correlations, alongside their ^13^C cross peaks at δ 118.0 ppm and 132.8 ppm were, assigned for tyrosine (M11) (Figure S6). Tryptophan (M12) was characterized from its indole moiety two triplet signals at δ 7.05 and 6.66 (t, *J* = 7.5 Hz), two doublets at δ 7.37 ppm and 7.69 ppm (d, *J* = 7.5 Hz), in addition to a singlet at δ 7.20 ppm, showing HSQC cross-peak correlation with ^13^C at δ 121.3, 117.4, 113.7, 120.5, and 126.5, respectively (Figure S6).

#### 2.4.4. Bufadienolides

A key feature of bufadienolides assignment in ^1^H-NMR spectra are signals of *α, β*-unsaturated ketone of pyranone ring at δ 6.26, 7.93 ppm showing total correlation at δ 7.41 ppm, showing HSQC cross-peak with ^13^C at 116.7, 150.5 and 151.8 ppm, respectively. Quaternary carbons C-20 and C-23 at δ 125.9 and 164.8 ppm, respectively were identified from by HMBC distinct correlation and aiding in their assignment. A major bufadienolide “bufalin” (M13) was detected in both squill identified from CH signals at C-17 δ 2.54, C-5 2.16, and C-3 3.84 ppm showing HSQC cross-peak correlation with ^13^C at δ 42.5, 54.2 and 72.4 ppm, respectively (Figures S2, S5 and S6). Two methyl groups at δ 0.74 and 0.98 ppm with ^13^C at δ 18.5 and 23.2 ppm alongside a quaternary C-14 appearing at ^13^C δ 85.4 in HMBC spectrum (Figure S3), respectively. Furthermore, “scilliridin” (M14) another bufadienolide was recognized from two conjugated double bonds at δ 5.71, 5.75 and 5.88 ppm showing HSQC cross-peak correlation with ^13^C at 128.5, 129.3, and 129.8 ppm, respectively, (Figure S6). Further distinct HMBC cross peaks identified C-5 at δ 141.3 ppm (Figure S7).

#### 2.4.5. Coumarins and Flavonoids

Hydroxy coumarin (M16) was identified based on *α-* and *β*-unsaturated ketone with signals at 6.18 and 7.85 ppm (d, *J* = 7.9 Hz), showing HSQC cross-peak correlations at 113.60 and 147.30 ppm, respectively (Figure S6). Moreover, an ABX benzene ring was revealed from signals at δ 6.71(d, *J* = 2.3 Hz), 6.80 (dd, *J* = 8.5, 2.3 Hz), and an overlapped peak at 7.4 ppm, showing HSQC cross-peak correlation at δ 104.63, 115.76, and 131.94 ppm, respectively (Figure S7).

Flavonoids were predicted primarily in RS and in accordance with UPLC-MS results are shown in Figure 2 and Table 2. An AABB system on chemical shift 6.98 (d, *J* = 8.5 Hz, 2H), 6.56 (d, *J* = 8.4 Hz, 2H), and chroman ring as AB system, *m*-position were elucidated from δH 6.04 (d, *J* = 1.8 Hz, 0H), and 6.04 (d, *J* = 1.8 Hz, H) was assigned to dihydrokaempferol (M15), (Figure S8).

### 2.5. Quantification of Major Metabolites via ^1^H-NMR

To aid in standardization of squill extract, ^1^H-NMR was further used to determine absolute levels of major metabolites in squill varieties via integration of their well-resolved signals in NMR spectra [57]. The concentration of metabolites was calculated as µg/mg dry powder, as shown in Supplementary Material Table S1.

With regard to primary metabolites, unsaturated fatty acids were detected at much higher levels in WS at 89.6 ± 25.3 versus 2.84 ± 0.5 µg/mL in RS. In contrast, a comparable amino acid level was detected in both varieties exemplified by aspartic acid as major form at 43.1 ± 7.2 and 38.3 ± 4.9 µg/mL in WS and RS, respectively. Other less abundant amino acids, including glycine, alanine, and tryptophan, were detected at 19.8 ± 3.5; 8.5 ± 1.6; and 2.2 ± 0.18 µg/mL in WS versus lower levels in RS at 7.2 ± 1.0; 0.3 ± 0.1; and 1.0 ± 0.1 µg/mL in RS, respectively. Sugars were found at comparable levels in both WS and RS at 42.5 ± 1.2 and 36.6 ± 0.5 µg/mL, respectively.

With regard to secondary metabolites to influence squill health effects, higher levels of total bufadeinolides distinguished by *α* and *β*-unsaturated ketone of pyranone ring (H-22) were measured in WS at 17.5 ± 7.5 µg/mL while in RS, dihydrokampferol was predominated at 43.6 ± 2.3 µg/mL. Finally, coumarins detected at almost equal levels in both WS and RS at ca. 5–6 µg/mg.

### 2.6. Cytotoxic Screening Activity

**Figure 8:**
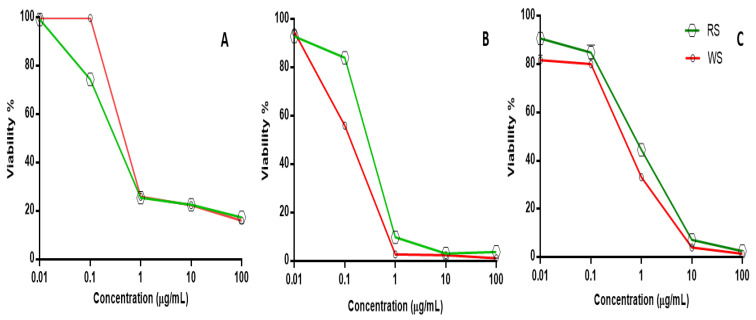
Cytotoxicity screening viability percentile versus RS and WS concentration (µg/mL) on different cancer cell lines. (**A**) MCF7 breast adenocarcinoma, (**B**) A-549 lung, and (**C**) SKOV-3 ovarian cancer cell lines.

Squill is recognized for its anticancer effect against various cancer cells and for its antioxidant and cytotoxic properties [58]. Consequently, a comparative cytotoxic assay of both WS and RS was evaluated on the different cell lines to include breast adenocarcinoma (MCF-7), lung (A-549) and ovarian cancer (SKOV-3) cell lines using Sulforhodamine B assay (SRB). The results revealed the potential cytotoxic activity of both varieties, with WS found to be more active against both cell lines A-549 and SKOV-3 than RS, as evidenced by its lower IC_50_ values. The recorded IC_50_ values of WS against A-549 and SKOV-3 cell lines were at 0.108 ± 0.003 and 0.690 ± 0.018 µg/mL versus RS’s IC_50_ values at 0.271 ± 0.005 and 0.912 ± 0.021 µg/mL, respectively. In contrast, the RS extract showed a more potent effect than WS on the MCF-7 cell line, with IC_50_ values of 0.165 ± 0.007 and 0.326 ± 0.005 µg/mL, respectively. The doxorubicin “positive control” IC_50_ values recorded on MCF7, A-549, and SKOV-3 cancer cell lines were 0.2 ± 0.004, 0.56 ± 0.003 and 0.2 ± 0.01 µg/mL, respectively (Table 4 and Figure 8).

The potential cytotoxicity of WS compared to RS could be attributed to its abundance of bufadienolides, as revealed from both LC/MS and NMR reported for its cytotoxic action, and suggests that bufadieonolides are more determinant than flavonoids regarding cytotoxic action in squill, at least in case of A-549 and SKOV-3 cell lines.

Previous reports of hellebrigenin and bufatalin isolated from squill showed potential cytotoxic activity against leukemia, human colon carcinoma, human glioblastoma melanoma, and human liver carcinoma cells with IC_50_ values ranging from 0.0007 to 0.16 μM [59]. Moreover, bufatalin induced apoptosis in human leukemia cells [60]. Scillarenin exhibited stronger cytotoxic action in the nanomolar range in comparison with bufatalin [61].

Proscillaridin A, a cardiac glycoside isolated from *U. maritima*, was reported to exert an cytotoxic and/or antiproliferative effect against human breast cancer. proscillaridin A anticancer properties are mediated via its ability to block Na+/K+ ATPase, leading to increase in Ca^2+^ levels, activating the AMPK pathway. Interestingly, on the opposite, the Ca^2+^ level was reduced by ca. 30% after an 18 h administration of proscillaridin A to the normal lung fibroblast cell line CCD19-LU, which suggests differential action mechanisms against normal and cancer cells [62].

In a previous report, bufadienolides recorded effective cytotoxic action on human cancer cells [63]. Although in WS, the most abundant bufadienolide glycoside “scilliroside” is suggested to mediate the observed toxic action of *Urginea* sp. The lethal dose (LD_50_) of scilliroside was 0.7 and 0.43 mg/kg for male and female rats in vivo, respectively. Scilliroside and its aglycon, scillirosidin, exert more toxic effect than other bufadienolides, such as proscillaridin and desacetylscillirosidin. This would be attributed for the presence of an acetoxy group at the C-6 position of scilliroside [63].

## 3. Materials and Methods

### 3.1. Plant Material

Samples of *U. maritima* (Linn) *Baker* “Sea Squill” (RS and WS) were collected in summer 2018–2019 from the El-Arish desert, Sinai, Egypt. RS and WS were authenticated by Prof. Zaki Turki (Department of Botany, Faculty of Science, El-Menoufia University, Shebin El-Kom, Egypt) as *U. maritima* (L.) *Baker*, and voucher specimens Sp. No HRE139 have been deposited at the Herbarium of the Department of Botany, Faculty of Science, Menoufia University, Egypt.

### 3.2. Secondary Metabolites Extraction and Preparation of NMR and MS Analysis Sample

The extraction protocol for NMR and MS analysis followed that by Farag et al. [14]. Briefly, a freeze-dried squill sample was mixed with 5 mL methanol with umbelliferone “internal standard (10 μg/mL) for the quantification of metabolites using UPLC/MS”. The squill extract was vortexed and then centrifuged (3000× *g*) for half an hour to remove any plant wastes. NMR analysis was performed by 3 mL aliquot, then concentrated under “N_2_” stream. The dried squill extract was resuspended with CD_3_OD (700 μL) containing 0.94 mM HMDS, then centrifugation at 13,000× *g* for 1 min.

### 3.3. SPME/GC-MS

Solid phase micro extraction (SPME) technique was adopted for volatiles extraction as in Farag et al. [64]. Squill (5 g) was incubated at 50 °C for half an hour in a screw cap glass vial, through which the SPME fibers “stableflex fibers covered with divinylbenzene/carboxen/polydimethylsiloxane (DVB/CAR/PDMS, 50/30 μm), Supelco (Oakville, ON, Canada)” was placed for 15 min with the squill sample, then injected into the GC-MS injection port. The GC-MS specifications and the analysis method were previously explained in detail in a previous study by Farag et al. [64].

### 3.4. UHPLC/MS

The identification of secondary metabolites in the squill sample was following the specifications of the UPLC/MS as well as the procedure that was previously mentioned in Farag et al. [65]. Characterization of the secondary metabolites was carried by their UV-VIS spectra from 200–600 nm, the retention time (Rt.) relative to authentic, exact mass and upon comparing the mass spectra of those authentic, the natural products database dictionary (CRC), and the published literature [66].

#### Multivariate Data Analysis of UPLC-MS & GC-MS Dataset

Metabolites of *U. maritima* were identified using UHPLC-MS/MS-Orbitrap-HRMS. The quantification was followed what was mentioned in Farag et al. [65]. In brief, the quantification was done using XCMS analysis software downloaded from (http://137.131.20.83/download/, accessed on 14 March 2023) [67,68]. The data was then analyzed using both PCA and OPLS-DA (SIMCA-P 13.0 software package-Umetrics, Umea, Sweden).

### 3.5. Identification of Major Metabolites via NMR Analysis

All spectra were analyzed using VNMRS 600 NMR spectrometer. All the specifications were described in detail as mentioned in Farag et al. [69]. The 2D-NMR spectra were reported at 599.83 MHz frequency using CHEMPACK 6.2 pulse sequences as COSY, HSQC, and HMBC. The optimization of HSQC and HMBC experiments were documented previously in [68]. Similar to the previous work [15], WS was used as a reference to demonstrate the identification of *U. maritima* metabolites. Interpretation was achieved by chemical shifts of standards using 2D-NMR and ^1^H-^1^H-correlation spectroscopy COSY and TOCSY, ^1^H-^13^C-HSQC, and HMBC.

#### Quantification of Major Metabolites via ^1^H-NMR

16 metabolites were quantified by NMR spectroscopy Figure S1. The peak area of both target compounds and the internal standard (HMDS) specific protons were interpreted manually for both squill samples as described in [69].

### 3.6. Bioassays

#### 3.6.1. Cell Culture

Different cancer cell lines have been tested, *viz.* breast adenocarcinoma (MCF-7), lung cancer (A-549), and ovarian cancer cells (SKOV-3). All cancer cells have been purchased from Nawah Scientific Inc., (Mokatam, Cairo, Egypt). Cells were kept in DMEM media supplemented with streptomycin (100 mg/mL), penicillin 100 (units/mL), and 10% of heat-inactivated fetal bovine serum in humid 5% (*v*/*v*) carbon dioxide at 37 °C.

#### 3.6.2. Cytotoxic Screening Assay

The cell viability was conducted by SRB assay. A 100 μL of cell suspension aliquots (5 × 10^3^ cells) was placed in 96-well plates, then incubated for 1 day. 100 μL media with squill extracts (0.01, 0.1, 1, 10, 100 µg/mL) were added to the cell suspension. After 3 days of treatment, the cells were fixed by changing the media with 150 μL (10% Trichloroacetic acid (TCA)) and incubated at 4 °C for 1 h. The cells were then washed with distilled water. SRB (70 μL) aliquots (0.4% *w*/*v*) were added and incubated in a dark place for 10 min. The plates were washed with acetic acid (1%) and allowed to dry overnight. Then, 150 μL of tris (hydroxymethyl) aminomethane (TRIS) (10 mM) was added, and absorbance was measured at 540 nm using a BMG LABTECH^®^-FLUOstar Omega microplate reader (Biotechnology company in Ortenberg, Germany).

#### 3.6.3. Statistical Analysis

The cytotoxic screening results were represented as averages of 3 independent experiments with their standard deviation (mean ± SD). For statistical significance determination, results were analyzed using one-way analysis of variance (ANOVA) followed by Dunnett’s post hoc test to compare doxorubicin positive control with treatment groups of WS and RS extracts on different cancer cell lines tested in vitro using SRB assay. Graph pad prism version 5 was used where *p* was ≤ 0.05.

## 4. Conclusions

In this current study, the two squill varieties (red and white) were investigated using a metabolomic approach (PCA and OPLS-DA) coupled with different chromatographic (SPME-GC/MS and UPLC/MS) and spectroscopic techniques (1D and 2D-NMR) for the first time, where metabolites diversity was identified in the two varieties.

Volatiles assessment (SPME-GC/MS) resulted in identifying 27 volatiles in both red and white squills. Red squill was enriched with monoterpenes hydrocarbons (47.82%) than white one (25.90%), loading to more aroma profiling. The 1D and 2D-NMR spectroscopic technique was utilized to identify 16 major metabolites. The phenolic compounds were abundant in the red squill, whereas bufadienolides and fatty acids showed more intense peaks in the white one.

Secondary metabolites identification (UHPLC/MS) revealed 130 metabolites representing a myriad of classes. Bufadienolides class was the major one in white squill, whereas flavonoids were the major one in the red variety. The 1D and 2D-NMR spectroscopic technique was utilized to further identify 16 major metabolites. The phenolic compounds were abundant in the red squill, whereas bufadienolides and fatty acids showed more intense peaks in the white one.

Multivariate data analysis differentiated between both varieties and confirmed the abundance of flavonoids in red squill exemplified in dihydrokaempferol-*O*-hexoside, its aglycon, and taxifolin derivative, whereas fatty acids (oleic and linoleic acids) in addition to bufadienolides, *viz.* hydroxyscilliglaucosidin-*O*-rhamnoside, desacetylscillirosidin-*O*-rhamnoside, and bufotalidin-*O*-hexoside are more abundant in the white one. A cytotoxicity screening was implemented on both squills against different cell lines revealed the effectiveness of white squill over red one due to its enrichment with bufadienolides class, which has yet to be confirmed using isolated bufadienolides to be conclusive.

## Figures and Tables

**Table 1:** Volatiles percentile of WS and RS using SPME coupled with GC-MS (n = 1).

Peak #	Rt (min)	KI	Name	Abundance %		Cas-No
				WS	RS	
Monoterpene hydrocarbon						
G1	7.13	909.1	*α*-Pinene	2.09	8.04	7785-26-4
G2	8.12	965.6	*β*-Myrcene	6.74	8.73	123-35-3
G4	8.46	985.3	3-Carene	2.32	7.81	13466-78-9
G5	8.47	985.6	2-Thujene	2.32	7.29	28634-89-1
G6	8.75	1002.4	*p*-Cymene	1.96	_	99-87-6
G7	8.81	1005.9	Limonene	4.89	7.81	5989-27-5
G8	9.27	1035.2	*α*-Phellandrene	1.72	_	99-83-2
G9	9.70	1063	Isoterpinolene	1.91	8.15	586-63-0
G10	9.71	1063.3	4-Carene	1.74	_	29050-33-7
Total monoterpene hydrocarbon				25.69	47.82	
Oxygenated monoterpene						
G15	11.47	1182.9	Estragole	2.29	_	140-67-0
Total oxygenated monoterpene				2.29		
Sesquiterpene hydrocarbon						
G21	15.12	1471.1	*β*-Bisabolene	8.22	_	495-61-4
Total sesquiterpene hydrocarbon				8.22		
Oxygenated hydrocarbon						
G12	10.03	1084.1	Nonanal	2.64	_	124-19-6
G14	11.44	1181.4	Decanal	2.49	_	112-31-2
G26	21.50	1841.9	Palmitic acid, methyl ester	7.02	_	112-39-0
Total oxygenated hydrocarbon				12.15		
Aromatic						
G19	14.79	1444.4	2-Methyl-4-hydroxyacetophenone	1.75	_	875-59-2
Total aromatic				1.75		
Heterocyclic						
G3	8.17	968.8	Furan-2-pentyl-	4.57	_	3777-69-3
Total heterocyclic				4.57		
Hydrocarbon						
G11	9.81	1069.5	Decane	2.64	6.31	124-18-5
G13	11.22	1166	Tridecane	4.92	6.03	629-50-5
G16	12.16	1235.5	4,7-Dimethylundecane	2.84	5.97	17301-32-5
G20	14.87	1451	Pentadecane	5.97	6.43	544-76-3
G24	17.72	1644	Hexadecane	_	7.29	544-76-3
G25	19.83	1754.6	Heptadecane	3.39	5.22	629-50-5
Total hydrocarbon				19.76	37.26	
Unknown						
G17	12.52	1261.8	Unknown	4.78	6.66	_
G18	13.73	1357.6	Unknown	18.11	_	_
G22	15.23	1480.8	Unknown	1.86	_	624-24-8
G23	16.13	1546	Unknown	_	8.27	_
Total unknown				24.955	14.93	
Total volatiles				100.0	100.0	

**Table 2:** Secondary metabolites tentatively identified in two squill varieties (WS and RS) via UPLC/MS in positive (L) and negative (N) ionization modes.

Code	t_R_ (m.)	UV	Elemental Composition	Error ppm	Exact Mass (M+H)^+^/ (M−H)^−^	Identified Fragments (MS^2^)	Identified Metabolite	Class	WS	RS
L1	0.4	280–300	(C_21_H_17_O_6_)⁺	8	365.1049	203, 185	Unknown glycoside	Unknown	+	−
L2	0.4	280–300	(C_27_H_27_O_11_)^+^	3.7	527.1567	365, 347, 203, 185	Unknown Glycoside (dihexose)	Unknown	+	−
L3	0.4	280–300	(C_21_H_31_O_12_)^+^	6.5	475.1779	133, 116	Hydroxycinnamyl-*O*-dihexoside	Phenolic	+	+
L4	0.4	280–300	(C_12_H_21_O_10_)^+^	2.9	325.112	145, 127	Disacharide (cellobiosan)	Sugar	+	+
L5	0.5	280–300	(C_33_H_37_O_16_)^+^	5	689.2041	527, 365, 347, 203, 185	Unknown glycoside (dihexoside)	Unknown	+	−
L6	0.6	280	(C_18_H_31_O_15_)^+^	2.2	487.1668	325, 271, 163, 145, 127	Agarotriose	Sugar	+	+
L7	0.6	280	(C_24_H_41_O_20_)^+^	4.4	649.2157	325, 289, 271, 253, 223,163, 145, 127	Polysaccharide (agarotriose dimer)	Sugar	−	+
L8	0.7	280	(C_9_H_12_NO_3_)^+^	3.1	182.0806	165, 147, 136, 123	Tyrosine	Amino acid	+	+
L9	1.2	280	(C_9_H_12_NO_2_)^+^	6.2	166.0852	120, 103	Phenylalanine	Amino acid	+	+
L10	1.6	280	(C_11_H_16_NO_2)_^+^	2.6	194.1171	148	Amino acid derivative	Amino acid	−	+
N1	1.7	280	(C_14_H_19_O_8_)^−^	3.4	315.1075	153	Isorahmantin	Flavonol	−	+
L11	2.3	280	(C_11_H_10_NO_2_)^+^	4.6	188.0697	170, 146	Tryptophan derivative	Amino acid	−	+
L12	2.5	280	(C_11_H_13_N_2_O_2_)⁺	6.1	205.0959	188, 170, 146, 118	Tryptophan	Amino acid	+	+
L13	2.8	282	(C_15_H_15_O_7_)^+^	−1.8	307.0818	289, 271, 261, 243, 188, 151, 139	Leucocyanidin		−	+
N2	2.9	280	(C_20_H_19_O_10_)^−^	0.7	419.0988	165	Juglanin	Flavonoid	−	+
N3	3.1	282–340	(C_21_H_23_O_11_)^−^	1.9	451.1246	289, 271, 243, 227, 199, 177, 151	Catechin-*O*-hexoside	Flavanol	−	+
L14	3.3	283–340	(C_15_H_15_O_6_)^+^	1.6	291.0859	273, 245, 151, 139	Catechin	Flavanol	−	+
N4	3.3	283–340	(C_21_H_23_O_11_)^−^	1.9	451.1246	271, 243, 227, 199, 177, 151	Catechin-*O*-hexoside isomer	Flavanol	−	+
L15	3.5	283–340	(C_15_H_11_O_6_)^+^	1.1	287.0547	259, 231, 149	Kaempferol	Flavone	−	+
N5	3.6	282–296–340	(C_20_H_27_O_14_)^−^	0	491.1406	209, 191, 167, 123	Vanillic acid-*O*-dihexoside	Phenolic acid	−	+
L16	3.7	282–296–340	(C_27_H_33_O_16_)^+^	5.1	613.1746	271, 243, 215	Dihydrokaempeferol-*O*-dihexoside	Flavanol	−	+
N6	3.8	282–296–340	(C_27_H_31_O_16_)^−^	1.6	611.1608	287, 269, 259, 243, 215	Dihydrokaempeferol-*O*-dihexoside (aromadendrin-*O*-dihexoside)	Flavanol	−	+
N7	3.9	282–296–340	(C_33_H_41_O_21_)^−^	0.4	773.2142	287, 269, 259, 243, 215	Dihydrokaempeferol-*O*-trihexoside (aromadendrin-*O*-trihexoside)	Flavanol	−	+
L17	4.2	291–330	(C_15_H_13_O_6_)^+^	−0.9	289.0709	271, 253, 243, 215, 149	Dihydrokaempferol	Flavanol	−	+
N8	4.2	291–330	(C_36_H_33_O_18_)^−^	0.3	753.167	465, 437, 315, 303, 285, 259, 245, 219	Dihydroquercitin-*O*-hexosidederivative (taxifolin-*O*-hexoside derivative)	Flavanol	−	+
L18	4.3	291–330	(C_21_H_23_O_11_)^+^	2.2	451.1245	289, 271, 247	Dihydrokaempferol-*O*-hexoside	Flavanol	−	+
L19	4.3	291–330	(C_38_H_45_O_25_^)+^	1.8	901.2228	451, 289, 271, 243	Dihydrokaempferol-*O*-hexoside dimer	Flavanol	−	+
L20	4.3	291–330	(C_15_H_11_O_5_)^+^	−0.2	271.0601	243, 215, 149	Apigenin	Flavone	−	+
L21	4.3	291–330	(C_14_H_11_O4)^+^	2.2	243.0646	215, 149	Unknown	Unknown	−	+
N9	4.3	291–330	(C_42_H_43_O_22_)^−^	−5.8	899.2304	449, 342, 287, 259	Dihydrokaempeferol-*O*-hexoside dimer	Flavanol	−	+
N10	4.4	291–330	(C_21_H_21_O_11_)^−^	6.3	449.1122	287, 269, 259, 243, 225, 151	Dihydro kaempeferol-*O*-hexoside (aromadendrin-*O*-hexoside	Flavanol	−	+
L22	4.6	283–296–340	(C_15_H_13_O_7_)^+^	1.8	305.065	287, 259, 231, 153	Dihydroquercitin	Flavanol	−	+
N11	4.6	283–296–340	(C_37_H_45_O_26_)^−^	2.1	905.2205	450, 285, 261, 243, 191	Kampferol derivative	Flavonol	-	+
L23	4.7	283–296–340	(C_15_H_11_O_8_)^+^	0	319.0812	301, 273, 269, 245	Myricetin	Flavonol	−	+
L24	4.7	283–296–340	(C_16_H_15_O_7_)^+^	−0.3	319.0813	313, 307, 286, 273, 245, 217, 185, 149, 137	Dihydroisorhmentin or Methyltaxifolin	Flavonoid	−	+
N12	4.6	283–296–340	(C_22_H_23_O_12_)^−^	2.9	479.1181	317, 306, 299, 289	Noidesol A/B	Flavonoid	−	+
L25	4.8	283–296-340	(C_20_H_29_O_13_)^+^	1	477.1598	169, 151	Vanillin rhamnoglucoside	Phenolic acid	−	+
	4.9	283–296-340	(C_20_H_27_O_13_)^−^	0.6	475.1457	209, 167, 123	Vanillin rhamnoglucoside	Phenolic acid	−	+
L26	4.8	283–296-340	(C_8_H_9_O_4_)^+^	2.8	169.049	151, 127, 109	Vanillic acid	Phenolic acid	−	+
N13	4.8	283–296-340	(C_15_H_11_O_6_)^−^	0.9	287.0559	201, 125	Dihydrokaempeferol	Dihydroflavonol	−	+
L27	4.9	283–296-340	(C_14_H_19_O_9_)^+^	4.8	331.1008	169	Vanillic acid-*O*-hexoside	Phenolic acid	−	+
N14	4.9	283–296-340	(C_21_H_29_O_12_)^−^	1.9	473.1651	209, 191, 167, 123	Dimethoxy hydrocinnamic acid (dimethoxyphenyl propionic acid)-dipentoside	Phenolic acid	−	+
L28	5.1	282	(C_21_H_23_O_10_)^+^	3.2	435.1272	271, 255, 151, 119	(Apigenin-*O*-hexoside)	Flavanone	−	+
L29	5.1	282	(C_9_H_7_O_3_)^+^	2.7	163.0385	135, 119, 107	Hydroxycoumarin	Coumarin	−	+
L30	5.1	282	(C_42_H_37_O_15_)^+^	1.3	781.2117	409, 317, 287, 247, 169	Unknown glycoside of L31	Unknown	−	+
N15	5.1	280	(C_9_H_5_O_3_)^−^	3.7	161.0244	133, 117, 105	Hydroxycoumarin	Coumarin	−	+
N16	5.4	283–340	(C_21_H_21_O_12_)^−^	0.7	465.1035	285, 275, 259, 231, 217, 152, 125	Kaempferol-*O*-hexoside	Flavonol	−	+
L31	5.5	283–340	(C_36_H_27_O_10_)^+^	3.6	619.1621	329, 317, 287, 271, 247, 229, 181, 169	Unknown aglycone of L30	Unknown	−	+
N17	5.5	283–340	(C_42_H_43_O_24_)^−^	4.4	931.2208	465, 303, 285, 275, 259, 231, 217, 152, 125	Dihydroquercitin-*O*-hexoside (dimer)	Flavanol	−	+
N18	5.6	283–340	(C_27_H_31_O_17_)^−^	0.2	627.1544	303,285, 217, 189, 151, 125	Dihydroquercitin-*O*- dihexoside	Flavanol	−	+
N19	5.6	283–340	(C_29_H_29_O_15_)^−^	0.1	617.1497	314, 285, 221, 209, 167, 125	Kampferol derivative	Flavanol	−	+
L32	5.7	283–340	(C_27_H_31_O_17_)^+^	6.3	627.1517	303	Quercitin-*O*-dihexoside	Flavonol	−	+
N20	5.7	283–340	(C_27_H_29_O_17_)^−^	0.2	625.1411	463, 301, 125	Quercitin-*O*-dihexoside	Flavonol	−	+
L33	5.9	283–340	(C_27_H_31_O_15_)^+^	6.7	595.1617	415, 397, 379, 361, 271	Apigenin-*O*-dihexoside	Flavanone	−	+
L34	6	299	(C_24_H_27_O_4_)^+^	0.5	379.1902	351, 333, 315, 239	Monohydroxy-19-oxobufa-4,20,22-trienolide	Bufadienolide	+	−
L35	6.1	299	(C_24_H_29_O_5_)^+^	0.3	397.2	381, 363, 345, 317	3-Dehydroscilliglaucosidin (scilliglaucosidine)	Bufadienolide	+	−
L36	6.2	299	(C_30_H_41_O_11_)^+^	1.1	577.2637	417, 399, 381, 363, 345, 335	Desacetylscillirosidin-*O*-thevetoside	Bufadienolide	+	+
N21	6.2	280–298-340	(C_15_H_11_O_7_)^−^	2.6	303.0502	294, 207, 181, 154, 99, 51	Dihydroquercitin	Flavanol	−	+
N22	6.3	280–298-340	(C_27_H_31_O_15_)^−^	2.3	595.1654	271, 151, 125	Naringenin-*O*-dihexoside	Flavanoid	−	+
N23	6.3	280–298-340	(C_21_H_19_O_12_)^−^	1.7	463.08	301, 300, 271, 151	Quercitin-*O*-hexoside	Flavonol	−	+
L37	6.5	299	(C_24_H_31_O_6_)^+^	2	415.2107	397, 379, 351, 333	Trihydroxy-oxobufa-trienolide (hydroxy-scilliglaucosidin)	Bufadienolide	+	−
L38	6.6	298	(C_30_H_45_O_10_)^+^	0.1	565.3008	403, 385, 367, 331, 272	Gammabufotalin*-O*-glucoside	Bufadienolide	+	−
L39	6.6	298	(C_24_H_35_O_5_)^+^	3	403.247	385,367, 253	Gamabufotalin	Bufadienolide	+	+
L40	6.6	280–298–340	(C_15_H_11_O_7_)^+^	0.8	303.05	191	Quercitin	Flavonol	−	+
L41	6.6	280–298–340	(C_21_H_21_O_12_)^+^	2.8	465.1015	308, 303	Quercitin-*O*-hexoside	Flavonol	−	+
L42	6.8	298	(C_30_H_43_O_10_)^+^	1.2	563.2844	545, 365, 347, 337, 323, 267, 252, 213	Scillirubroside	Bufadienolide	+	−
L43	6.9	298	(C_24_H_31_O_4_)⁺	2.8	383.2228	365, 348, 251	Scillirubrosidin-H_2_O(scillirubroside-hexoside-H_2_O)	Bufadienolide	+	+
L44	7	298	(C_24_H_29_O_6_)⁺	1.2	413.1954	395, 69, 351, 333	Trihydroxy-oxobufa-tetra-enolide	Bufadienolide	+	−
L45	7	298	(C_43_H_45_O_11_)^+^	2.2	737.294	413, 395, 377, 359, 331	Trihydroxy-oxobufa-tetra-enolide-*O*-di-hexoside	Bufadienolide	+	−
L46	7.2	299	(C_30_H_41_O_11_)^+^	3.8	577.2621	415, 397, 379, 351, 333	Hydroxyscilliglaucoside	Bufadienolide	+	+
N24	7.7	280–298–340	(C_21_H_19_O_11_)^−^	3.7	447.0916	285, 255, 227	Kaempferol-*O*-glucoside (hexoside)	Flavonol	−	+
L47	7.8	299	(C_36_H_51_O_15_)^+^	8.1	723.3164	561, 415, 397, 379, 361	Trihydroxy-oxobufa-trienolide-*O*-rhamnosdie-glucoside/Scilliglaucosidin-*O*-rhamnoside-glucoside	Bufadienolide	+	−
L48	7.9	299	(C_24_H_33_O_6_)^+^	1.8	417.2279	399, 381, 363, 345, 335, 145	Desacetylscillirosidin/Hydroxyscilliphaeosidin/Bufotalidin	Bufadienolide	+	−
L49	7.9	299	(C_24_H_33_O_6_)^+^	1.8	417.2279	399, 381, 363, 335,	Desacetylscillirosidin/Hydroxyscilliphaeosidin/Bufotalidin isomer	Bufadienolide	+	−
L50	8	299	(C_30_H_41_O_10_)⁺	1.1	561.269	415, 379, 361, 351	Trihydroxy-oxobufa-trienolide-*O*-rhamoside/Hydroxy-scilliglaucosidin-*O*-rhamnoside	Bufadienolide	+	+
N25	8.1	280–298–340	(C_22_H_21_O_12_)^−^	1.9	477.103	315, 314, 299, 285, 271, 243	Isorhamnetin 3-hexoside	Flavonol	−	+
L51	8.3	299	(C_30_H_43_O_10_)⁺	2.1	563.2839	417, 399, 381, 363, 345, 315, 278	Desacetylscillirosidin-*O*-rhamnoside/Hydroxyscilliphaeosidin-*O*-rhamnoside	Bufadienolide	+	−
L52	8.3	299	(C_30_H_43_O_10_)⁺	2.1	563.2839	417, 399, 381, 363, 333	Bufotalidin-*O*-rhamnoside	Bufadienolide	+	−
L53	8.5	299	(C_24_H_31_O_5_)^+^	0.9	399.2169	381, 363, 345, 223.,157	Scilliglaucosidin	Bufadienolide	+	+
L54	8.7	298	(C_26_H_37_O_8_)^+^	0.1	477.248	417, 399, 381, 363, 345	Hydroxy-scillirosidin+2H	Bufadienolide	+	−
L55	8.8	298	(C_30_H_45_O_9_)^+^	0.8	549.3053	403, 385, 367, 349, 193, 179	Gamabufotalin-*O*-rhamnoside	Bufadienolide	+	−
L56	8.9	298	(C_24_H_33_O_5_)^+^	1.9	401.2315	383, 347, 197	Scillirubrosidin or scilliphosidin	Bufadienolide	+	+
L57	8.9	298	(C_30_H_43_O_9_)^+^	2.9	547.2886	401,383, 347	Scilliphaeoside “Scillipheosidin-*O*-rhamnoside”	Bufadienolide	+	+
L58	8.9	299	(C_36_H_53_O_14_)^+^	2.6	709.3411	547, 417, 367, 349, 287	Scillipheoside*-O*-glucoside	Bufadienolide	+	+
L59	8.9	285	(C_34_H_27_O_12_^)+^	0.1	627.1498	401, 383, 365, 303, 269, 193	Scillirubrosidin-*O*-hexoside or scilliphosidin-o-hexoside	Bufadienolide	−	+
N26	8.9	299	(C_36_H_67_O_26_)^−^	1.5	915.3912	869, 707, 545, 399, 355, 221, 161, 113	Unknown	Unknown	−	+
L60	9.3	283	(C_36_H_55_O_14_)^+^	4.6	711.3554	549, 531, 403, 367, 349, 253, 199	Gamabufotalin-rhamnoglucoside	Bufadienolide	−	+
L61	9.6	298	(C_24_H_27_O_3_)⁺	2.5	363.1946	345, 335, 317, 273	Unknown	Unknown	+	−
L62	10	299	(C_30_H_41_O_10_)⁺	1.1	561.269	399, 381, 363, 345, 223, 157	Scilliglaucoside	Bufadienolide	+	+
L63	10	399	(C_33_H_49_O_12_)^+^	2.7	637.3201	477, 417, 399, 381, 363, 345	Hydroxyscilliroside	Bufadienolide	+	−
L64	10	299	(C_24_H_31_O_3_)^+^	4.1	367.2253	349, 287, 175, 133	Scillaridin A	Bufadienolide	+	+
L65	11	298	(C_39_H_59_O_17_)^+^	7.2	799.3651	477, 399, 381, 363, 345	Hydroxyscilliphaeosidin-*O*-thevetoside-glucoside-Ac	Bufadienolide	+	−
L66	11	282	(C_36_H_45_O_19_)^+^	4.5	781.2514	325, 241, 163, 145, 115	Unknown	Unknown	−	+
L67	12	298	(C_33_H_55_O_8_)^+^	3.1	579.387	417, 237, 255	Bufotalidin-*O*-hexoside	Bufadienolide	+	+
L68	12	298	(C_30_H_41_O_9_)⁺	4.6	545.272	399, 381, 363, 353, 345, 335, 317	Scilliglaucosidin-*O*-rhmnoside	Bufadienolide	+	−
L69	12	299	(C_36_H_51_O_14_)^+^	8.4	707.3214	545,399, 381, 363, 335, 317, 275, 223	Scilliglaucosidin-*O*-rhmnoside-*O*-hexoside	Bufadienolide	+	−
L70	12	299	(C_24_H_33_O_4_)^+^	0	385.2373	367, 349, 289, 253	Scillarenin	Bufadienolide	+	+
L71	12	283	(C_24_H_29_O_3_)^+^	−1.9	365.2118	349, 287, 175, 147	Unknown aglycone of L42	Bufadienolide	−	+
L72	12	296	(C_26_H_35_O_6_)^+^	2.9	443.2415	425, 383, 365, 347, 319, 269, 239, 225, 197	Cinobufagin or acetylmarinobufogenin	Bufadienolide	−	+
L73	12	296	(C_45_H_51_O_10_)^+^	0.6	751.3472	589, 443, 425, 365, 347, 285, 225, 173	Cinobufagin or acetylmarinobufogenin-*O*-rhamnoside-glucoside	Bufadienolide	−	+
L74	12	299	(C_17_H_29_O_7_)^+^	0.4	345.1906	281, 263, 253, 193	Unknown	Unknown	+	−
L75	12	283	(C_19_H_38_NO_5_)^+^	3.8	360.2731	342, 324, 306, 278, 260, 240, 222	3-Hydroxydodecanoylcarnitine	Acylcarnitine	−	+
L76	12	298	(C_33_H_49_O_11_)^+^	3.2	621.3249	461, 401, 383, 365, 319, 251, 213	Scilliroside	Bufadienolide	+	−
L77	12	296	(C_32_H_45_O_11_)^+^	5.8	605.2921	591, 572, 537, 529, 462, 443, 417, 337, 256, 237, 207, 165, 145, 135, 108	Cinobufagin-*O*-hexoside or acetylmarinobufogenin-*O*-hexoside	Bufadienolide	−	+
L78	12	298	(C_24_H_35_O_6_)^+^	4.5	419.2409	401, 383, 365, 347, 213	Hellebrigenol (19-hydroxytelocinobufagin)	Bufadienolide	+	−
L79	12	298	(C_38_H_57_O_16_)^+^	5.3	769.36	607, 461, 401 383, 365, 347, 305	Scillirosidin-rhamnoside-glucoside+2H	Bufadienolide	+	−
L80	12	299	(C_39_H_65_O_13_)⁺	12.8	741.4325	579, 461, 419, 401, 383, 365	Hellebrigenin-3-*O*-D-diglucopyranoside.	Bufadienolide	+	+
L81	12	300	(C_38_H_59_O_19_)^+^	-1.4	819.3692	367, 349, 273, 255, 237	Unknown	Unknown	−	+
L82	12	299	(C_24_H_35_O_4_)^+^	7.6	387.25	385, 367, 349, 331, 199	Bufalin	Bufadienolide	−	+
L83	12	300	(C_30_H_45_O_8_)^+^	1.4	533.3102	515, 387, 367, 349, 274, 255, 199	Dihydro-Proscillaridin (rhamnosylbufalin)	Bufadienolide	−	+
L84	12	298	(C_39_H_59_O_16_)⁺	5.3	783.3697	621, 543, 461, 401, 383, 365, 347,251	Scilliroside-*O*-glucoside	Bufadienolide	+	−
L85	12	299	(C_38_H_61_O_20_)^+^	1.3	837.3761	515, 387, 349, 255, 237	Bufalin derivative	Bufadienolide	−	+
L86	12	299	(C_36_H_53_O_13_)^+^	4	693.3394	531, 385, 367, 349, 287	Proscillaridin A-*O*-glucoside	Bufadienolide	+	+
L87	12	299		7	362.2876	344, 308, 224	Unknown fat	Fat	−	+
L88	12	299	(C_26_H_37_O_7_)^+^	3.3	461.2549	401, 383, 365, 337, 329, 305, 285	Dihydroscillirosidin	Bufadienolide	+	−
L89	12	298	(C_38_H_55_O_14_)^+^	10.7	735.351	573, 461, 385, 367, 349, 331, 287	Scillaren A acetate-*O*-rhamnoside-hexoside	Bufadienolide	+	−
L90	12	299	(C_32_H_47_O_11_)⁺	3.4	607.3092	547, 401, 383, 365, 347	Acetyl-scilliphaeoside-rhamnoside	Bufadienolide	+	−
L91	13	298	(C_30_H_43_O_8_)^+^	1.7	531.2943	513, 385, 367, 349, 321, 303, 253, 215	Proscillaridin A	Bufadienolide	+	+
L92	13	298	C_44_H_65_O_19_	13.7	897.3992	735, 573, 385, 367, 349, 331, 287	Scillaren A acetate-*O*-rhamnoside-dihexoside	Bufadienolide	+	−
L93	13	282	(C_16_H_13_O_5_)^+^	4.9	285.0744	191	Acacetin or prunetin	Flavonoid	−	+
L94	13	282	(C_17_H_17_O_6_)^+^	2.8	317.1011	299, 271, 121	Dihydroxy-dimethoxyflavanone	Flavonoid	−	+
L95	13	286	(C_20_H_42_NO_6_)^+^	2.3	392.2997	356, 338, 278, 261, 232	*N*-tetradecyl-D-gluconamide	Amide	−	+
L96	13	280	(C_20_H_42_NO_5_)^+^	1.7	376.3051	340, 262, 245, 219	2-(14-Aminotetradecyl)-6-(hydroxymethyl) oxane-3,4,5-triol	Fatty alcohol	−	+
L97	13	280	(C_19_H_38_NO_4_)^+^	1.6	344.279	326, 308, 280, 224	19-(hydroxyamino)-19-oxo-nonadecanoic acid	Fatty acid	−	+
L98	13	280	(C_19_H_36_NO_3_)^+^	3.5	326.2678	308, 252	Dodecadienyl carnitine	Fatty acyl-L-carnitine	−	+
L99	13	280	(C_20_H_40_NO_4_)^+^	2.1	358.2944	340, 322, 294	Tridecanoyl carnitine	Fatty acyl-L-carnitine	−	+
L100	13	280	(C_18_H_40_NO_4_)^+^	3.7	334.294	316, 298, 280, 251, 238	1-(hydroperoxyamino) octadecane-1,18-diol	Fatty alcohol	−	+
L101	14	299	(C_32_H_45_O_9_)^+^	4.4	573.3033	367, 349, 331, 253, 133	Scillaridin-acetate-*O*-rhamnoside	Bufadienolide	+	−
L102	14	298	(C_16_H_27_O_4_)^+^	3.9	283.1893	270, 265	Fumagillol	Sesquiterpenoid	+	−
L103	14	298	(C_17_H_27_O_5_)^+^ (C16H27O3)+	2.1	311.1847 (−46 Formate)	265, 247, 209	(4E,6Z)-3-Hydroxy-4,6,15-hexadecatrienoic acid	Acid	+	−
L104	14	282	(C_19_H_40_NO_4_)^+^	5.1	346.2934	328, 310, 282, 264, 226	Monomethyl phytosphingosine	Sphingolipid	−	+
L105	14	298	(C_17_H_29_O_6_)^+^	3.7	329.1946	265, 237, 209, 191,	Spiculisporic acid	Acid	+	−
L106	14	282	(C_20_H_42_NO_4_)^+^	1.3	360.3104	342, 324, 296	*N*-acetyl phytosphingosine	Sphingolipid	−	+
L107	14	282	(C_17_H_17_O_5_)^+^	4.6	301.1057	282, 267	Unknown	Unknown	−	+
L108	14	289	(C_18_H_40_NO_3_)^+^	4.2	318.2989	300, 282, 270, 264	Phytosphingosine	Sphingolipid	−	+
L109	14	289	(C_19_H_21_O_5_)^+F^	2.5	329.1375	207, 121	Hirsutanone		−	+
L110	14	-	(C_30_H_23_O_10_)^+^	1.8	543.1296	273, 255, 213	Unknown	Unknown	+	−
L111	14	-	(C_18_H_40_NO_2_)⁺	4.7	302.3039	284, 266, 254	Sphinganine	Sphingolipid	+	+
L112	14	-	(C_17_H_31_O_5_)⁺	5.1	315.215	265	Unknown	Unknown	+	−
L113	14	280	(C_27_H_45_O_3_)^+^	2.3	417.3354	273, 255, 161	24-Hydroperoxycholesta-5,25-dien-3beta-ol	Fatty acid	−	+
L114	15	-	(C_18_H_31_O_2_)^+^	0.3	279.2318	261, 223, 173	Octadecatrienoic acid	Fatty acid	+	+
N27	15	-	(C_18_H_31_O_3_)^−^	2.2	295.2279	195, 277	Coriolic acid	Fatty acid	−	+
L116	16	-	(C_48_H_81_O_8_)⁺	11.5	733.4379	367,253	Dimer of unknown fatty acid	Fatty acid	+	+
L117	16	-	(C_18_H_29_O_3_)^+^	5.2	293.2096	275, 223, 95	Licanic acid	Fatty acid	+	−
L118	17	-	(C_21_H_39_O_4_)^+^	0.1	355.2843	337, 263	Glyceryl 2-linoleate	Fatty acid	+	−
N28	17	-	(C_18_H_29_O_2_)^−^	3.1	277.2164	250, 226, 171, 150, 109, 77, 53	Linolenic acid	Fatty acid	−	+
L119	17	-	(C_18_H_27_O)⁺	1.1	259.2054	175	Unknown	Unknown	+	−
L120	17	-	(C_18_H_33_O_2_)^+^	0.4	281.2476	263, 245, 189,	Linoleic acid	Fatty acid	+	+
L121	17	-	(C_20_H_33_O_3_)^+^	1.7	321.243	305, 265, 245, 179	8-Hydroxyicosa-5,9,11,14-tetraenoic acid	Fatty acid	+	−
L122	17	-	(C_36_H_65_O_4_)⁺	3.9	561.4899	543, 307, 245, 175	Linoleic acid dimer	Fatty acid	+	−
L123	17	-	(C_21_H_41_O_4_)^+^	5.4	357.298	339, 265, 247, 205, 135, 124, 112, 75	Glyceryl Monooleate	Fatty acid	+	+
N29	17	-	(C_18_H_31_O_2_)^−^	0.9	279.2327	201, 167, 141, 127, 89, 70, 54	Linoleic acid	Fatty acid	+	+
L124	17.7	-	(C_16_H_31_O)^+^	6.7	239.2353	109, 95	Hexadeca-10,12-dien-1-ol	Fatty acid	+	+
L125	17.7	-	(C_16_H_33_O_2_)^+^	3.3	257.2467	237, 120, 103	Hexadecanoic acid	Fatty acid	+	+
L126	17.8	-	(C_18_H_35_O_2_)⁺	5.9	283.2615	265, 247, 191, 153, 137, 121	Palmitic acid	Fatty acid	+	−
L127	17.8	-	(C_18_H_33_O)^+^	6.1	265.251	247, 205, 191, 149	Linolenyl alcohol	Fatty alcohol	+	+
N30	17.8	-	(C_18_H_33_O_2_)^−^	0.8	281.24	185, 155, 95, 58	Oleic acid	Fatty acid	+	+
N31	17.8	-	C19H35O4	10	327.2505	281, 185, 95	Chaetomellic acid A	Fatty acid	+	−
L128	18	-	(C_14_H_23_O_16_)^+^	1.6	447.0988	359, 341, 324, 225, 207, 149	Unknown	Unknown	−	+
L129	18	-	(C_22_H_39_O_4_)^+^	0.1	367.2843	349, 331,293, 251, 205, 179, 133	16,17-dihydroxydocosa-7,10,13-trienoic acid	Fatty acid	+	+
L130	18	-	(C_22_H_37_O_3_)^+^	2.6	349.2728	331, 293, 183, 165	Anacardic acid	Phenolic lipid	+	+

**Table 3:** Assignment data from 1D and 2D NMR on WS and RS extracts.

Metabolite	Assignment	δ ^1^H in ppm	δ ^1^H COSEY (ppm)	δ ^13^C in ppm	HMBC Correlations δ ^13^C in ppm
Fatty acid (M1–M3)	Olefinic carbons	5.23–5.34	2.05	128.0–131.3	29.4 (bis allylic CH_2_), 27.9 allylic CH_2_
	allylic CH_2_	2.07 m	5.34	27.9	Olefinic 128.0–131.0, (CH_2_)_n_ 31.5
ω-9 Fatty acid (M1)	t-CH_3_	0.91 (t, *J* = 6.9 Hz)	1.3 (CH_2_)n	14.1	23.9 C-2, 31.5(CH_2_)_n_
	(CH2)n	1.3 (br. s)	0.89 (t-CH3), 1.61 (H-3), 2.09 (allylic CH2)	30.4	32.0 (CH_2_)_n_
	C-2	2.27 (t, *J* = 7.4 Hz)	1.59	34.7	C-1 178.8, C-32.4, (CH_2_)n 31.6
	C-3	1.59 m	2.27	25.8	C-1 177.8, (CH_2_)_n_ 30.4
ω-6 Fatty acid (M2)	Bis allylic CH_2_	2.77 (t, *J* = 6.9 Hz)	5.33	24.7	Olefinic carbons 131.2,
ω-3 Fatty acid (M3)	Bis allylic CH_2_	2.87	5.33	26.3	Olefinic carbons 131.2,
	t-CH_3_	0.97	2.07 (allylic CH_2_)	19.5	21.6 C-2
Sugars					
Rhamnoside (M4)	C-6	1.23	3.77	19.5	(C-4) 69.8, (C-5) 72.8
	C-1	5.44	-	93. 8	(C-2) 73.8
	C-5	3.77	-	72.7	-
	C-4	3.78	-	69.1	-
*β*-Glucose (M5)	C-1	4.48 (d, *J* = 7.8 Hz)	3.11, 3.27, 3.32	97.9	-
	C-2	3.11		76.1	(C-3) 77.8, (C-1)97.9
	C-3	3.35		77.8	-
	C-5	3.28		77.7	(C-6) 62.2
	C-6	3.65		62.1	-
*α*-Glucose (M6)	C-1	5.10 (d*, J* = 3.7 Hz)	3.35	93.7	(C-4) 72.7, (C-3)74.61
	C-2	3.35		73.5	74.2 (C-3)
	C-3	3.84		74.2	-
	C-4	3.77		72.7	(C-6) 62.5
	C-6	3.86		62.5	-
Sucarose (M7)	C-1	5.48 (t, *J* = 3.6 Hz)	3.35, 3.42, 3.69	93.3	(C-3) 74.3, (C-1′) 105.0
	C-2	3.42	3.69	72.9	(C-3) 74.3
	C-3	3.69	3.35	74.2	(C-2) 72.9
	C-4	3.35		72.8	(C-3) 74.3
	C-2′	4.08	-	87.9	(C-1′) 105.0
	C-3′	4.03	-	76.8	(C-5′) 63.1, (C-1′) 105.0
	C-4′	3.75	-	83.6	(C-6′) 63.9
	C-5′	3.76	-	63.1	(C-1′) 105.0, (C-4′) 83.6
	C-6′	3.62	-	63.9	(C-1′) 105.0
Amino acids					
Alanine (M8)	C-3	1.47 (d, *J* = 7.2)	3.64	16.9	51.5, 174.9
	C-2	3.64	1.47	51.4	-
Aaspartic acid (M9)	C-3a	2.96	2.71, 3.85	35.3	(C-2) 52.6, (C-4) 172.9, (C-1)174.9
	C-3b	2.71	-	35.3	(C-2) 52.6, (C-4) 172.9, (C-1)174.9
	C-2	3.85	-	52.6	(C-4) 172.9, (C-1) 174.9
Glycine (M10)	C-2a	3.88	-	43.6	
	C-2b	4.01	-	43.6	(C-1) 174.7
Tyrosine (M11)	C-3	2.98–3.31		40.0	(C-4) 127.2
	C-2	4.14		50.6	
	C-6, C-8	6.77	7.13	116.5	(C-4) 127.2
	C-5, C-9	7.13	6.77	131.3	(C-7) 157.6
Tryptophan (M12)	C-2	3.89	-	56.5	(C-3) 24.9, (C-1) 173.4
	C-8	7.05 (t, *J* = 7.5 Hz)	7.12, 7.37	119.8	(C-7) 112.2, (C-11) 128.3
	C-9	7.12 (overlap)	7.69	122.5	(C-6) 138.2, (C-8) 119.0
	C-5	7.20 (s)	-	124.9	(C-4) 102.1, (C-6) 138.2
	C-10	7.69 (d, *J* = 7.9 Hz)	7.37	118.9	(C-9) 122.5, (C-6)138.2
	C-7	7.37 (d, *J* = 8.2 Hz)	-	112.2	-
Bufadienolides					
Bufalin and Scilliridin (M13, M14)	C-21	7.98 (d, *J* = 2.6 Hz)	6.25	149.9	
	C-22	6.27 (d, *J* = 3.0 Hz)	7.97	115.2	122.4, 164.3 (C-20, 23)
	C-24	7.43 (s)		150.3	122.4, 164.2 (C-20, 23)
	C-17	2.59		51.7	124.2 (C-20)
	C-8	1.83	1.68	42.8	84.0, 49.0 (C-14, 13)
	C-9	1.68	1.83	43.1	
Bufalin (M13)	C-18	0.714		12.1	(C-13) 49.0, (C-17) 51.0, (C-14) 84.0
	C-19	0.98		19.0	(C-1) 35.0, (C-2) 37.0, (C-9) 57.0
	C-3	3.93		73.5	(C-1) 35.0
Scilliridin (M14)					
	C-3	5.96		128.6	(C-5)141.0
	C-4	5.75		127.3	-
	C-6	5.71		127.5	(C-5) 141.0
	C-19	1.02 3H		19.2	(C-10) 38.3, (C-9) 51.9, (C-6) 141.4
	C-18	0.81		17.7	(C-13) 49.0, (C-17) 51.0, (C-14) 84.0
Flavanoids					
kaempeferol derv. (M15)	C (3′, 5′)	δ 6.98 (d, *J* = 8.5 Hz, 2H)		-	-
	C (2′, 6′)	6.56 (d, *J* = 8.4 Hz, 2H)		-	-
	C-8	6.04 (d, *J* = 1.8 Hz, H)		-	-
	C-6	6.04 (d, *J* = 1.8 Hz, H)		-	-
Coumarins					
6-Hydroxy coumarin (M16)	C-3	6.19 (d, *J* = 9.4 Hz)	7.84	112.1	(C-10) 112.9, 157.0
	C-4	7.84 (d, *J* = 9.4 Hz)	6.19	145.8	(C-2) 157.0
	C-8	6.72 (d, *J* = 2.3 Hz)		103.1	(C-6) 157.6
	C-6	6.80 (dd, *J* = 8.5, 2.3 Hz)	7.47	114.25	(C-7) 154
	C-5	7.47 overlap (d, *J* = 8.5 Hz)	6.80	130.43	(C-4) 145.79

**Table 4:** The IC_50_ values (mean ± SD) of RS and WS extract against different cancer cell lines measured by SRB assay versus dox. (doxorubicin) positive control.

RS (MCF7) IC50	WS (MCF7) IC50	Dox.(MCF7) IC50	RS (A-549) IC50	WS (A-549) IC50	Dox. (A-549) IC50	RS (SKOV-3) IC50	WS (SKOV-3) IC50	Dox. (SKOV-3)IC50
0.165 ± 0.007 *	0.326 ± 0.005	0.2 ± 0.004 *	0.271 ± 0.005 *	0.108 ± 0.003 *	0.56 ± 0.003 *	0.912 ± 0.021	0.690 ± 0.018	0.2 ± 0.01

* *p* < 0.05 comparing dox. positive control with each treatment group of RS and WS extracts on different cancer cell lines using one way ANOVA followed by Dunnett’s test.

## Data Availability

Data are available from omarkhattab500@gmail.com upon request.

## Supplementary Materials

The following supporting information can be downloaded at https://www.mdpi.com/article/10.3390/plants12112078/s1, Figure S1: ESI-MS/MS spectrum of metabolites in the positive ion mode; Figure S2: Signal assignment of the ^1^H-NMR markers for fatty acids (M1–M3), rhamnose (M4), alanine (M8), aspartic acid (M9), glycine (M10), and bufadienolides (M13 & M14) using ^1^H-^13^C correlations observed in the HSQC spectrum of squill methanol extract; Figure S3: Signal assignment of the ^1^H-NMR markers for fatty acids (M1-M3), rhamnose (M4), alanine (M8), aspartic acid (M9), glycine (M10), and bufadienolides (M13&M14) using ^1^H-^13^C correlations observed in the HMBC spectrum of squill methanol extract; Figure S4: Signal assignment of the ^1^H-NMR markers for fatty acids (M1-M3), rhamnose (M4), β, α glucose (M5 & M6), sucrose (M7), aspartic acid (M9), glycine (M10), tryptophan (M12), and bufalin (M13) using ^1^H-^13^C correlations observed in the HMBC spectrum of squill methanol extract; Figure S5: Signal assignment of the ^1^H-NMR markers for rhamnose (M4), β, α glucose (M5& M6), sucrose (M7), aspartic acid (M9), glycine (M10), tryptophan (M12), and bufalin (M13) using ^1^H-^13^C correlations observed in the HSQC spectrum of squill methanol extract; Figure S6: Signal assignment of the ^1^H-NMR markers for tyrosine (M11), tryptophan (M12), bufalin (M13), scilliridin (M14), dihydro kaempferol (M15), and coumarin (M16); ^1^H-^13^C correlations observed in the HSQC spectrum of squill methanol extract; Figure S7: Signal assignment of the ^1^H-NMR markers for tyrosine (M11), tryptophan (M12), bufalin (M13), scilliridin (M14), dihydro kaempferol (M15), and coumarin (M16); ^1^H-^13^C correlations observed in the HMBC spectrum of squill methanol extract; Figure S8: Signal assignment of the proton markers for dihydro kaempferol (M15) observed in the ^1^H-NMR spectrum of RS methanol extract; Table S1: ^1^H-NMR quantification of most common primary and secondary metabolites detected in Urginea species, white squill (WS) and red squill (RS). Values are expressed as μg/mg dry powder ± S.D (n = 3). Chemical shifts used for metabolite quantification were determined in methanol-d_6_ and expressed as relative values to HMDS (0.94 mM final concentration); Table S2: RS and WS extract inhibition and viability values in different concentrations against different cancer cell lines measured by SRB assay.

## References

[1] Bozorgi M., Amin G., Shekarchi M., Rahimi R. (2017). Traditional medical uses of Drimia species in terms of phytochemistry, pharmacology and toxicology. J. Tradit. Chin. Med..

[2] El-Seedi H.R., Burman R., Mansour A., Turki Z., Boulos L., Gullbo J., Goransson U. (2013). The traditional medical uses and cytotoxic activities of sixty-one Egyptian plants: Discovery of an active cardiac glycoside from *Urginea maritima*. J. Ethnopharmacol..

[3] BayazıT V., Konar V. (2010). Analgesic Effects of Scilliroside, Proscillaridin-A and Taxifolin from Squill Bulb (*Urginea maritima*) on Pains. Dig. J. Nanomater. Biostruct. (DJNB).

[4] Dizaye K., Hamed B. (2010). Cardiovascular studies of white squill (*Urginea maritima*) extract. Zanco J. Med. Sci..

[5] Leblanc F.J., Lee C.O. (1939). A study of the toxic principles of red squill. J. Am. Pharm. Assoc..

[6] Santos C.V.d., Kerkhoff J., Tomazelli C.A., Wenceslau C.F., Sinhorin A.P., de Jesus Rodrigues D., Carneiro F.S., Bomfim G.F. (2022). Vasoconstrictor and hemodynamic effects of a methanolic extract from Rhinella marina toad poison. Toxicon.

[7] Wang H.-Y.L., O’Doherty G.A. (2012). Modulators of Na/K-ATPase: A patent review. Expert Opin. Ther. Pat..

[8] Aswal S., Kumar A., Semwal R.B., Chauhan A., Kumar A., Lehmann J., Semwal D.K. (2019). Drimia indica: A plant used in traditional medicine and its potential for clinical uses. Medicina.

[9] Wu C.-H., Tao W., Yamaguchi Y., Yue C., Han L.-F., Zhang Y. (2013). A new phenylpropanol glycoside and its five known analogues from *Boschniakia rossica*. Chin. Herb. Med..

[10] Metin M., Bürün B. (2010). Effects of the high doses of *Urginea maritima* (L.) baker extract on chromosomes. Caryologia.

[11] Bozorgi M., Amin G., Kasebzade S., Shekarchi M. (2016). Development and validation of a HPLC-UV method for determination of Proscillaridin A in *Drimia maritima*. Res. J. Pharmacogn..

[12] Singh V., Soni L.K., Dobhal S., Jain S.K., Parasher P., Dobhal M.P. (2016). Phytochemicals and Pharmacological Properties of Urginea Species. Chem. Sci. Rev. Lett..

[13] Krenn L., Kopp B., Steurer S., Schubert-Zsilavecz M. (1996). 9-Hydroxyscilliphaeoside, a new bufadienolide from *Urginea maritima*. J. Nat. Prod..

[14] Rasheed D.M., Porzel A., Frolov A., El Seedi H.R., Wessjohann L.A., Farag M.A. (2018). Comparative analysis of *Hibiscus sabdariffa* (roselle) hot and cold extracts in respect to their potential for α-glucosidase inhibition. Food Chem..

[15] Worley B., Powers R. (2016). PCA as a practical indicator of OPLS-DA model reliability. Curr. Metab..

[16] Okada T., Mochamad Afendi F., Altaf-Ul-Amin M., Takahashi H., Nakamura K., Kanaya S. (2010). Metabolomics of medicinal plants: The importance of multivariate analysis of analytical chemistry data. Curr. Comput.-Aided Drug Des..

[17] Jha S. (1988). Bufadienolides. Phytochemicals in Plant Cell Cultures.

[18] Feng W., Hao Z., Li M., Justino G.C. (2017). Isolation and Structure Identification of Flavonoids. Flavonoids, from Biosynthesis to Human Health.

[19] Barrueto F., Kirrane B.M., Cotter B.W., Hoffman R.S., Nelson L.S. (2006). Cardioactive steroid poisoning: A comparison of plant-and animal-derived compounds. J. Med. Toxicol..

[20] Iizuka M., Warashina T., Noro T. (2001). Bufadienolides and a new lignan from the bulbs of *Urginea maritima*. Chem. Pharm. Bull..

[21] Crouch N.R., du Toit K., Mulholland D.A., Drewes S.E. (2006). Bufadienolides from bulbs of *Urginea lydenburgensis* (Hyacinthaceae: Urgineoideae). Phytochemistry.

[22] Shimada K., Umezawa E., Nambara T., Kupchan S.M. (1979). Isolation and characterization of cardiotonic steroids from the bulb of *Urginea altissima* Baker. Chem. Pharm. Bull..

[23] Kopp B., Krenn L., Draxler M., Hoyer A., Terkola R., Vallaster P., Robien W. (1996). Bufadienolides from *Urginea maritima* from Egypt. Phytochemistry.

[24] Cao Y., Wu J., Pan H., Wang L. (2019). Chemical Profile and Multicomponent Quantitative Analysis for the Quality Evaluation of Toad Venom from Different Origins. Molecules.

[25] Mohamed G.A., Ibrahim S.R.M., Shaala L.A., Alshali K.Z., Youssef D.T.A. (2014). Urgineaglyceride A: A new monoacylglycerol from the Egyptian *Drimia maritima* bulbs. Nat. Prod. Res..

[26] Koorbanally N.A., Koorbanally C., Harilal A., Mulholland D.A., Crouch N.R. (2004). Bufadienolides from *Drimia robusta* and *Urginea epigea* (Hyacinthaceae). Phytochemistry.

[27] Wei W., Yu Y., Wang X., Yang L., Zhang H., Ji H., Li Z., Hou J., Wu W., Guo D. (2019). Simultaneous Determination of Bufalin and Its Nine Metabolites in Rat Plasma for Characterization of Metabolic Profiles and Pharmacokinetic Study by LC–MS/MS. Molecules.

[28] Kakouri E., Kanakis C., Trigas P., Tarantilis P.A. (2019). Characterization of the chemical composition of *Drimia numidica* plant parts using high-resolution mass spectrometry: Study of their total phenolic content and antioxidant activity. Anal. Bioanal. Chem..

[29] Knittel D.N., Stintzing F.C., Kammerer D.R. (2015). Metabolic fate of cardiac glycosides and flavonoids upon fermentation of aqueous sea squill (*Drimia maritima* L.) extracts. J. Pharm. Biomed. Anal..

[30] Bose C., Chakrabarty A. (2002). 4,5-Dihydro-14-[beta]-Hydroxy Scilladienolide-3-O-[beta]-D-Glucopyranoside (AC-3) from the Stems of *Milletia ovalifolia*. Asian J. Chem..

[31] Fang S., Tao H., Xia K., Guo W. (2019). Proscillaridin A induces apoptosis and inhibits the metastasis of osteosarcoma in vitro and in vivo. Biochem. Biophys. Res. Commun..

[32] Triana-Martínez F., Picallos-Rabina P., Da Silva-Álvarez S., Pietrocola F., Llanos S., Rodilla V., Soprano E., Pedrosa P., Ferreirós A., Barradas M. (2019). Identification and characterization of Cardiac Glycosides as senolytic compounds. Nat. Commun..

[33] Da Costa E.M., Armaos G., McInnes G., Beaudry A., Moquin-Beaudry G., Bertrand-Lehouillier V., Caron M., Richer C., St-Onge P., Johnson J.R. (2019). Heart failure drug proscillaridin A targets MYC overexpressing leukemia through global loss of lysine acetylation. J. Exp. Clin. Cancer Res..

[34] Yuan B., Shimada R., Xu K., Han L., Si N., Zhao H., Bian B., Hayashi H., Okazaki M., Takagi N. (2019). Multiple cytotoxic effects of gamabufotalin against human glioblastoma cell line U-87. Chem.-Biol. Interact..

[35] He R., Ma H., Zhou J., Zhu Z., Lv X., Li Q., Wang H., Yan Y., Luo N., Di L. (2019). High Resolution Mass Profile of Bufadienolides and Peptides Combing with Anti-Tumor Cell Screening and Multivariate Analysis for the Quality Evaluation of Bufonis Venenum. Molecules.

[36] Pohl T., Koorbanally C., Crouch N.R., Mulholland D.A. (2001). Bufadienolides from *Drimia robusta* and *Urginea altissima* (Hyacinthaceae). Phytochemistry.

[37] Krenn L., Stapf V., Kopp B. (2000). Bufadienolides from *Drimia robusta* BAK. Sci. Pharm..

[38] Pinheiro P.F., Justino G.C., Rao V. (2012). Structural analysis of flavonoids and related compounds—A review of spectroscopic applications. Phytochemicals—A Global Perspective of Their Role in Nutrition and Health.

[39] Ye M., Yang W.-Z., Liu K.-D., Qiao X., Li B.-J., Cheng J., Feng J., Guo D.-A., Zhao Y.-Y. (2012). Characterization of flavonoids in *Millettia nitida* var. hirsutissima by HPLC/DAD/ESI-MSn. J. Pharm. Anal..

[40] Ragab E.A., Raafat M. (2016). A new monoterpene glucoside and complete assignments of dihydroflavonols of *Pulicaria jaubertii*: Potential cytotoxic and blood pressure lowering activity. Nat. Prod. Res..

[41] Knittel D.N., Stintzing F.C., Kammerer D.R. (2014). Simultaneous determination of bufadienolides and phenolic compounds in sea squill (*Drimia maritima* (L.) Stearn) by HPLC-DAD-MS n as a means to differentiate individual plant parts and developmental stages. Anal. Bioanal. Chem..

[42] Fernandez M., Vega F.A., Arrupe T., Renedo J. (1972). Flavonoids of squill, *Urginea maritima*. Phytochemistry.

[43] Belhaddad O.E., Charef N., Amamra S., Zerargui F., Baghiani A., Khennouf S., Arrar L. (2017). Chromatographic fractionation, antioxidant and antibacterial activities of *Urginea maritima* methanolic extract. Pak. J. Pharm. Sci..

[44] March R.E., Lewars E.G., Stadey C.J., Miao X.-S., Zhao X., Metcalfe C.D. (2006). A comparison of flavonoid glycosides by electrospray tandem mass spectrometry. Int. J. Mass Spectrom..

[45] Olennikov D.N., Gadimli A.I., Isaev J.I., Kashchenko N.I., Prokopyev A.S., Kataeva T.N., Chirikova N.K., Vennos C. (2019). Caucasian Gentiana Species: Untargeted LC-MS Metabolic Profiling, Antioxidant and Digestive Enzyme Inhibiting Activity of Six Plants. Metabolites.

[46] Cui W., He Z., Zhang Y., Fan Q., Feng N. (2019). Naringenin Cocrystals Prepared by Solution Crystallization Method for Improving Bioavailability and Anti-hyperlipidemia Effects. AAPS PharmSciTech.

[47] Shimokawa Y., Akao Y., Hirasawa Y., Awang K., Hadi A.H.A., Sato S., Aoyama C., Takeo J., Shiro M., Morita H. (2010). Gneyulins A and B, stilbene trimers, and noidesols A and B, dihydroflavonol-C-glucosides, from the bark of *Gnetum gnemonoides*. J. Nat. Prod..

[48] Abbas S., Bashir S., Khan A., Mehmood M.H., Gilani A.H. (2012). Gastrointestinal stimulant effect of *Urginea indica* Kunth. and involvement of muscarinic receptors. J. Phytother. Res..

[49] Bashir S., Abbas S., Gilani A.H., Khan A. (2013). Studies on bronchodilator and cardiac stimulant activities of *Urginea indica*. J. Bangladesh J. Pharmacol..

[50] Kumar N., Goel N. (2019). Phenolic acids: Natural versatile molecules with promising therapeutic applications. Biotechnol. Rep..

[51] Méndez-Líter J.A., Tundidor I., Nieto-Domínguez M., de Toro B.F., Santana A.G., de Eugenio L.I., Prieto A., Asensio J.L., Sánchez C., Martínez M.J. (2019). Transglycosylation products generated by *Talaromyces amestolkiae* GH3 β-glucosidases: Effect of hydroxytyrosol, vanillin and its glucosides on breast cancer cells. Microb. Cell Factories.

[52] Seong Y.-A., Hwang D., Kim G.-D. (2016). The anti-inflammatory effect of *Gnaphalium affine* through inhibition of NF-κB and MAPK in lipopolysaccharide-stimulated RAW264.7 cells and analysis of its phytochemical components. Cell Biochem. Biophys..

[53] Jaakola L., Määttä K., Pirttilä A.M., Törrönen R., Kärenlampi S., Hohtola A. (2002). Expression of genes involved in anthocyanin biosynthesis in relation to anthocyanin, proanthocyanidin, and flavonol levels during bilberry fruit development. Plant Physiol..

[54] Emwas A.-H., Roy R., McKay R.T., Tenori L., Saccenti E., Gowda G.N., Raftery D., Alahmari F., Jaremko L., Jaremko M. (2019). NMR spectroscopy for metabolomics research. Metabolites.

[55] Kim H.K., Choi Y.H., Verpoorte R. (2010). NMR-based metabolomic analysis of plants. Nat. Protoc..

[56] Lin C.Y., Wu H., Tjeerdema R.S., Viant M.R. (2007). Evaluation of metabolite extraction strategies from tissue samples using NMR metabolomics. Metabolomics.

[57] Khattab A.R., Rasheed D.M., El-Haddad A.E., Porzel A., Wessjohann L.A., Farag M.A. (2022). Assessing phytoequivalency of four Zingiberaceae spices (galangals, turmeric and ginger) using a biochemometric approach: A case study. Ind. Crops Prod..

[58] Saket K., Afshari J.T., Saburi E., Yousefi M., Salari R. (2020). Therapeutic aspects of Squill; an evidence-based review. Curr. Drug Discov. Technol..

[59] Cunha-Filho G.A., Resck I.S., Cavalcanti B.C., Pessoa C.Ó., Moraes M.O., Ferreira J.R.O., Rodrigues F.A.R., dos Santos M.L. (2010). Cytotoxic profile of natural and some modified bufadienolides from toad *Rhinella schneideri* parotoid gland secretion. Toxicon.

[60] Tempone A.G., Pimenta D.C., Lebrun I., Sartorelli P., Taniwaki N.N., de Andrade Jr H.F., Antoniazzi M.M., Jared C. (2008). Antileishmanial and antitrypanosomal activity of bufadienolides isolated from the toad *Rhinella jimi* parotoid macrogland secretion. Toxicon.

[61] Mahringer A., Karamustafa S., Klotz D., Kahl S., Konkimalla V.B., Wang Y., Wang J., Liu H.-Y., Boechzelt H., Hao X. (2010). Inhibition of P-glycoprotein at the blood–brain barrier by phytochemicals derived from traditional Chinese medicine. Cancer Genom. -Proteom..

[62] Li R.-Z., Fan X.-X., Duan F.-G., Jiang Z.-B., Pan H.-D., Luo L.-X., Zhou Y.-L., Li Y., Yao Y.-J., Yao X.-J. (2018). Proscillaridin A induces apoptosis and suppresses non-small-cell lung cancer tumor growth via calcium-induced DR4 upregulation. Cell Death Dis..

[63] Manganyi M.C., Tlatsana G.S., Mokoroane G.T., Senna K.P., Mohaswa J.F., Ntsayagae K., Fri J., Ateba C.N. (2021). Bulbous Plants Drimia: “A Thin Line between Poisonous and Healing Compounds” with Biological Activities. Pharmaceutics.

[64] Farag M.A., Gad H.A., Heiss A.G., Wessjohann L.A. (2014). Metabolomics driven analysis of six Nigella species seeds via UPLC-qTOE-MS and GC-MS coupled to chemometrics. Food Chem..

[65] Farag M.A., El-Kersh D.M., Ehrlich A., Choucry M.A., El-Seedi H., Frolov A., Wessjohann L.A. (2019). Variation in Ceratonia siliqua pod metabolome in context of its different geographical origin, ripening stage and roasting process. Food Chem..

[66] Fabre N., Rustan I., de Hoffmann E., Quetin-Leclercq J. (2001). Determination of flavone, flavonol, and flavanone aglycones by negative ion liquid chromatography electrospray ion trap mass spectrometry. J. Am. Soc. Mass Spectrom..

[67] Maghraby Y.R. (2021). Nanoencapsulation of *Jania rubens*’ Phytochemicals: Antioxidant Properties for Food Applications. Ph.D. Thesis.

[68] Smith C.A., Want E.J., O’Maille G., Abagyan R., Siuzdak G. (2006). XCMS: Processing mass spectrometry data for metabolite profiling using nonlinear peak alignment, matching, and identification. Anal. Chem..

[69] Farag M.A., Khaled S.E., El Gingeehy Z., Shamma S.N., Zayed A. (2022). Comparative Metabolite Profiling and Fingerprinting of Medicinal Cinnamon Bark and Its Commercial Preparations via a Multiplex Approach of GC–MS, UV, and NMR Techniques. Metabolites.

