# Supplementary material for: Comparative MS- and NMR-Based Metabolome Mapping of Egyptian Red and White Squill Bulbs F. Liliaceae and in Relation to Their Cytotoxic Effect
Source: Plants (Basel). 2023 May 23;12(11):2078. doi: 10.3390/plants12112078 (PMC10255078; doi:10.3390/plants12112078)
Supplement: Supplementary file 1 [file plants-12-02078-s001.zip › plants-2313227-supplementary.pdf]

Table S1. ^{1}H-NMR quantification of most primary and secondary metabolites detected in *Urginea* species; white squill (WS) and red squill (RS). Values are expressed as µg/mg dry powder ± S.D (n = 3). Chemical shifts used for metabolite quantification were determined in methanol-d_6 and expressed as relative values to HMDS (0.94 mM final concentration).

compound	Protons used in quantification	WS	RS
**ω-9 fatty acid (M1)**	H-3 CH_2	57.52 ±25.342	1.2437 ±0.525
**ω-6 fatty acid (M2)**	bis-allylic CH_2	32.1697±13.82	1.6003 ±0.0177
**ω-3 fatty acid (M3)**			
**Rhamnose (M4)**	H-6 CH_3	10.4557 ±1.119	11.39 ±0.285
**β-glucose (M5)**	H-1	15.0467 ±0.602	8.2347 ±0.721
**α-glucose (M6)**	H-1	15.5767 ±1.19	8.767 ±0.474
**Sucrose (M7)**	H-1	1.4733 ±0.463	8.2263 ±0.43
**alanine (M8)**	H-3	8.5873 ±1.631	0.3513 ±0.133
**aspartic acid (M9)**	H-1	43.07 ±7.217	38.3367 ±4.943
**glycine (M10)**	H-2	19.79 ±3.534	7.1673 ±1.032
**Tyrosine (M11)**	H-6	3.964 ±0.66	-
**Tryptophan (M12)**	H-10	2.2853 ±0.18	1.0087 ±0.115
**bufalin (M13)**	H-22	17.4867 ±7.502	-
**Scilliradin (M14)**			
**dihydro kaempferol (M15)**	H-6'		43.61 ±2.318
**Coumarin (M16)**	H-4	5.9703 ±0.282	4.6927 ±0.282

Table S2. RS and WS extract inhibition and viability values in different concentrations against different cancer cell lines measured by SRB assay.

Conc.	RS (MCF7)							WS (MCF7)						
**µg/mL**	**Viability**	**Inhibition**	**Viability**	**Inhibition**	**Viability**	**Inhibition**	**MEAN±SD**	**Viability**	**Inhibition**	**Viability**	**Inhibition**	**Viability**	**Inhibition**	**MEAN±SD**
**0**	100	0	100	0	100	0		100	0	100	0	100	0	
**0.01**	99.291	0.709	97.48	2.52	101.1	-1.1		99.917	0.083	100	0	99.752	0.248	
**0.1**	74.567	25.433	74.409	25.591	74.488	25.512		99.089	0.911	100.5	-0.5	99.917	0.083	
**1**	24.882	75.118	25.433	74.567	26.063	73.937		25.828	74.172	26.325	73.675	25.414	74.586	
**10**	22.52	77.48	22.598	77.402	22.52	77.48		22.185	77.815	22.599	77.401	22.351	77.649	
**100**	17.48	82.52	17.323	82.677	17.008	82.992		14.983	85.017	16.06	83.94	16.887	83.113	
**IC50**		0.1636		0.1734		0.1593	**0.165±0.007**		0.326		0.3309		0.32	**0.326±0.005**
**Conc.**	**RS (A-549)**							**WS (A-549)**						
**µg/mL**	**Viability**	**Inhibition**	**Viability**	**Inhibition**	**Viability**	**Inhibition**	**MEAN±SD**	**Viability**	**Inhibition**	**Viability**	**Inhibition**	**Viability**	**Inhibition**	**MEAN±SD**
**0**	100	0	100	0	100	0		100	0	100	0	100	0	
**0.01**	92.965	7.035	94.831	5.169	91.098	8.902		94.79	5.21	94.588	5.412	94.992	5.008	
**0.1**	84.35	15.65	84.853	15.147	83.202	16.798		55.446	44.554	56.726	43.274	55.985	44.015	
**1**	9.9067	90.0933	10.122	89.878	9.6195	90.3805		2.8296	97.1704	2.2906	97.7094	3.3685	96.6315	
**10**	3.1587	96.8413	3.8765	96.1235	2.4408	97.5592		2.9643	97.0357	2.0885	97.9115	2.2232	97.7768	
**100**	3.8047	96.1953	3.6612	96.3388	3.9483	96.0517		1.0779	98.9221	1.2127	98.7873	1.4148	98.5852	
**IC50**		0.2727		0.2661		0.2756	**0.271±0.005**		0.1053		0.1115		0.1079	**0.108±0.003**
**Conc.**	**RS (SKOV-3)**							**WS (SKOV-3)**						
**µg/mL**	**Viability**	**Inhibition**	**Viability**	**Inhibition**	**Viability**	**Inhibition**	**MEAN±SD**	**Viability**	**Inhibition**	**Viability**	**Inhibition**	**Viability**	**Inhibition**	**MEAN±SD**
**0**	100	0	100	0	100	0		100	0	100	0	100	0	
**0.01**	92.074	7.926	90.415	9.585	89.585	10.415		81.267	18.733	79.89	20.11	84.022	15.978	
**0.1**	81.843	18.157	87.926	12.074	84.885	15.115		79.89	20.11	78.237	21.763	82.369	17.631	
**1**	45.069	54.931	43.963	56.037	44.516	55.484		33.196	66.804	31.956	68.044	34.435	65.565	
**10**	7.4654	92.5346	7.1889	92.8111	6.9124	93.0876		3.0303	96.9697	4.4077	95.5923	4.6832	95.3168	
**100**	2.4885	97.5115	2.765	97.235	2.4885	97.5115		1.1019	98.8981	1.6529	98.3471	1.6529	98.3471	
**IC50**		0.9077		0.8932		0.9357	**0.912±0.021**		0.7059		0.6708		0.6955	**0.690±0.018**

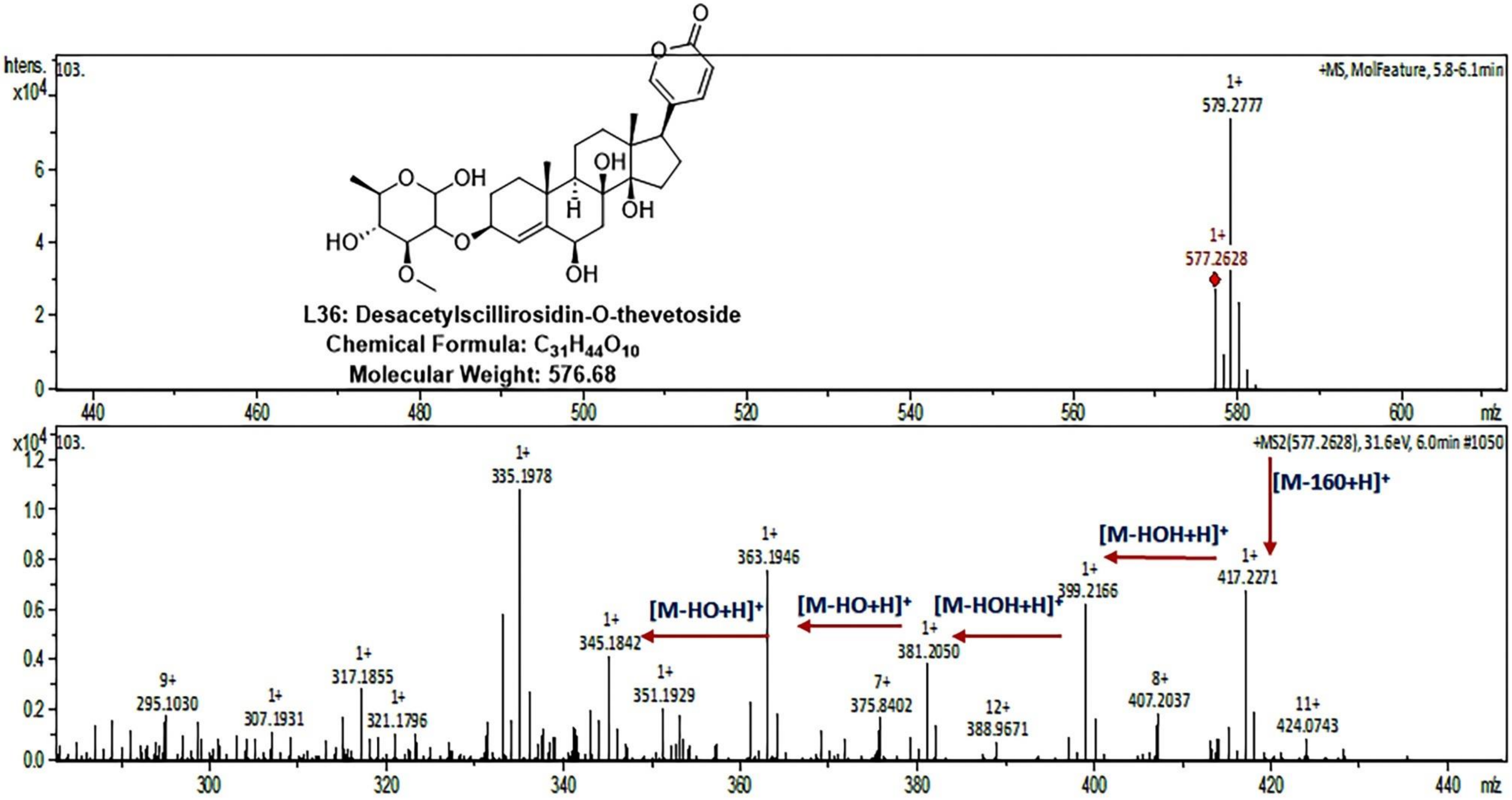

Figure S1.-A: ESI-MS/MS spectrum of L36 in the positive ion mode.

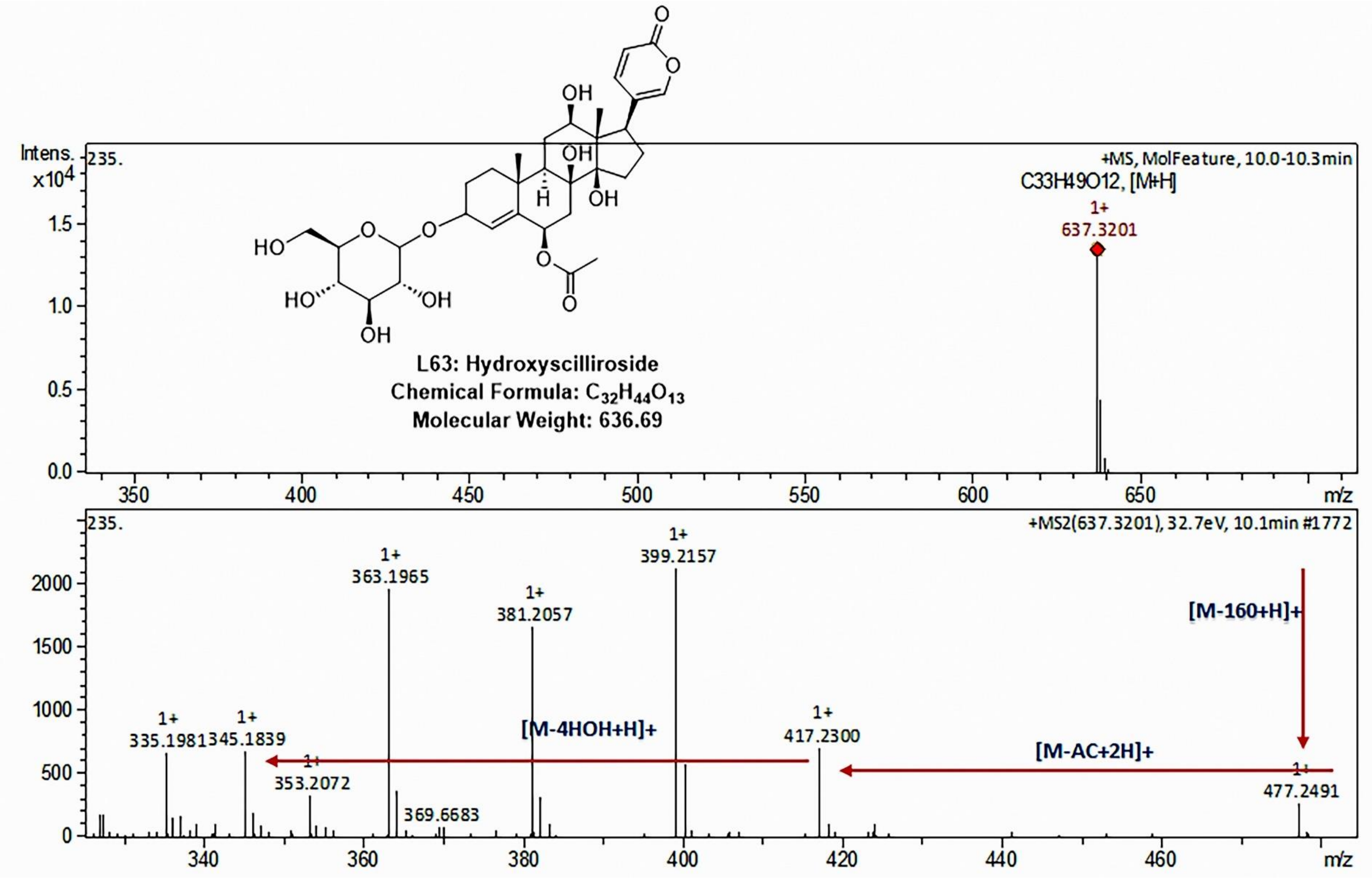

Figure S1.-B: ESI-MS/MS spectrum of L63 in the positive ion mode.

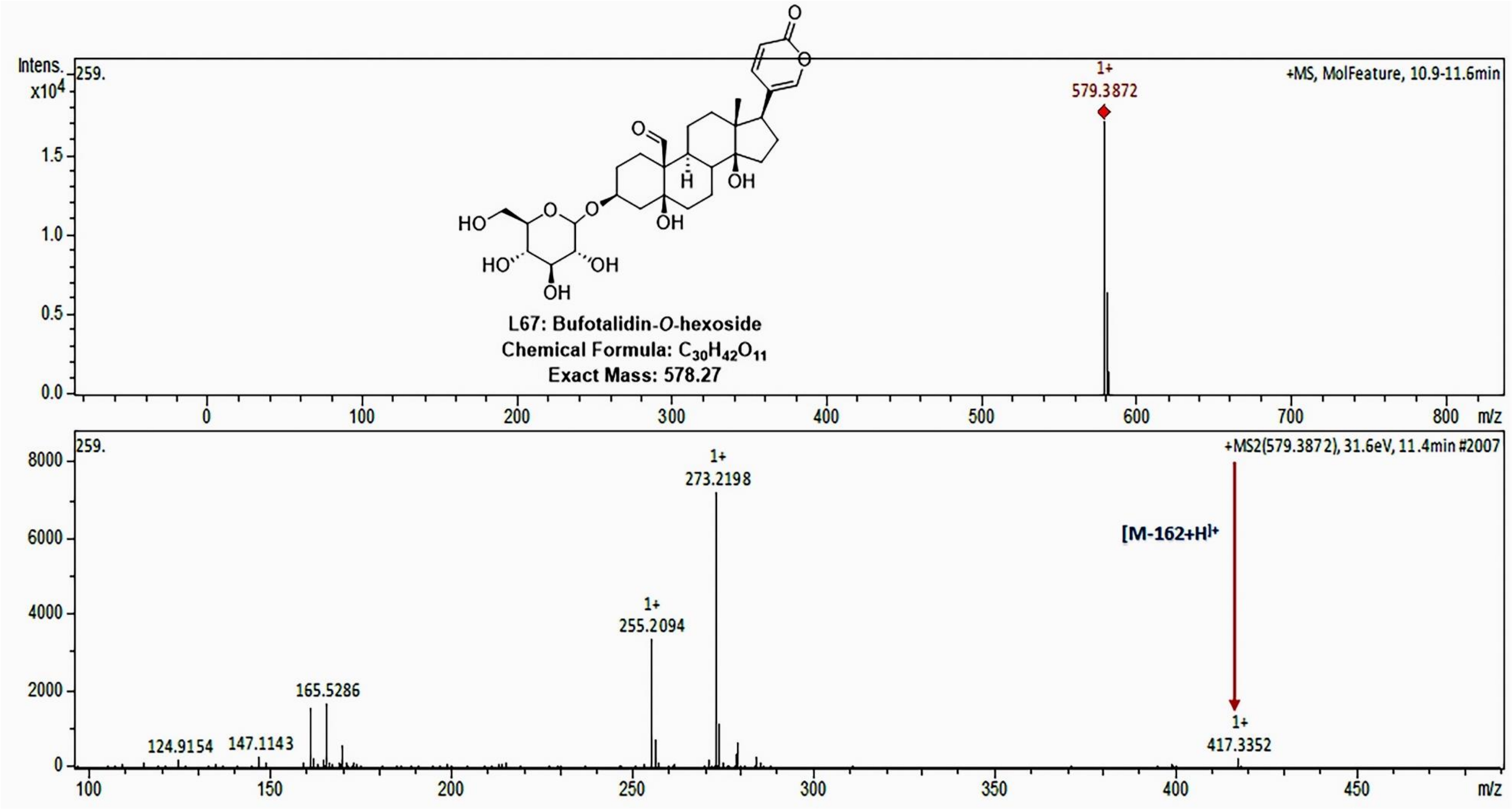

Figure S1.-C: ESI-MS/MS spectrum of L67 in the positive ion mode.

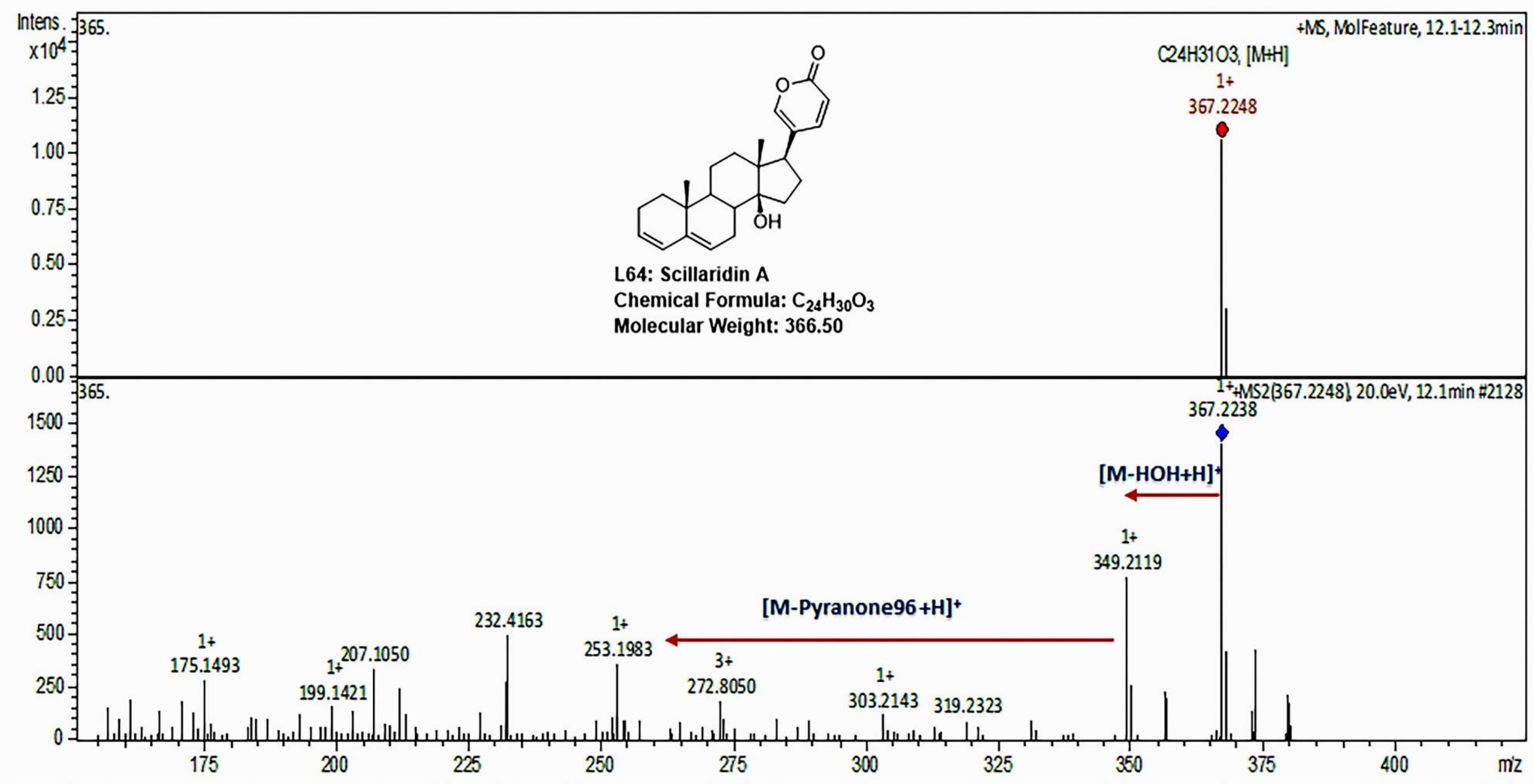

Figure S1.-D: ESI-MS/MS spectrum of L64 in the positive ion mode.

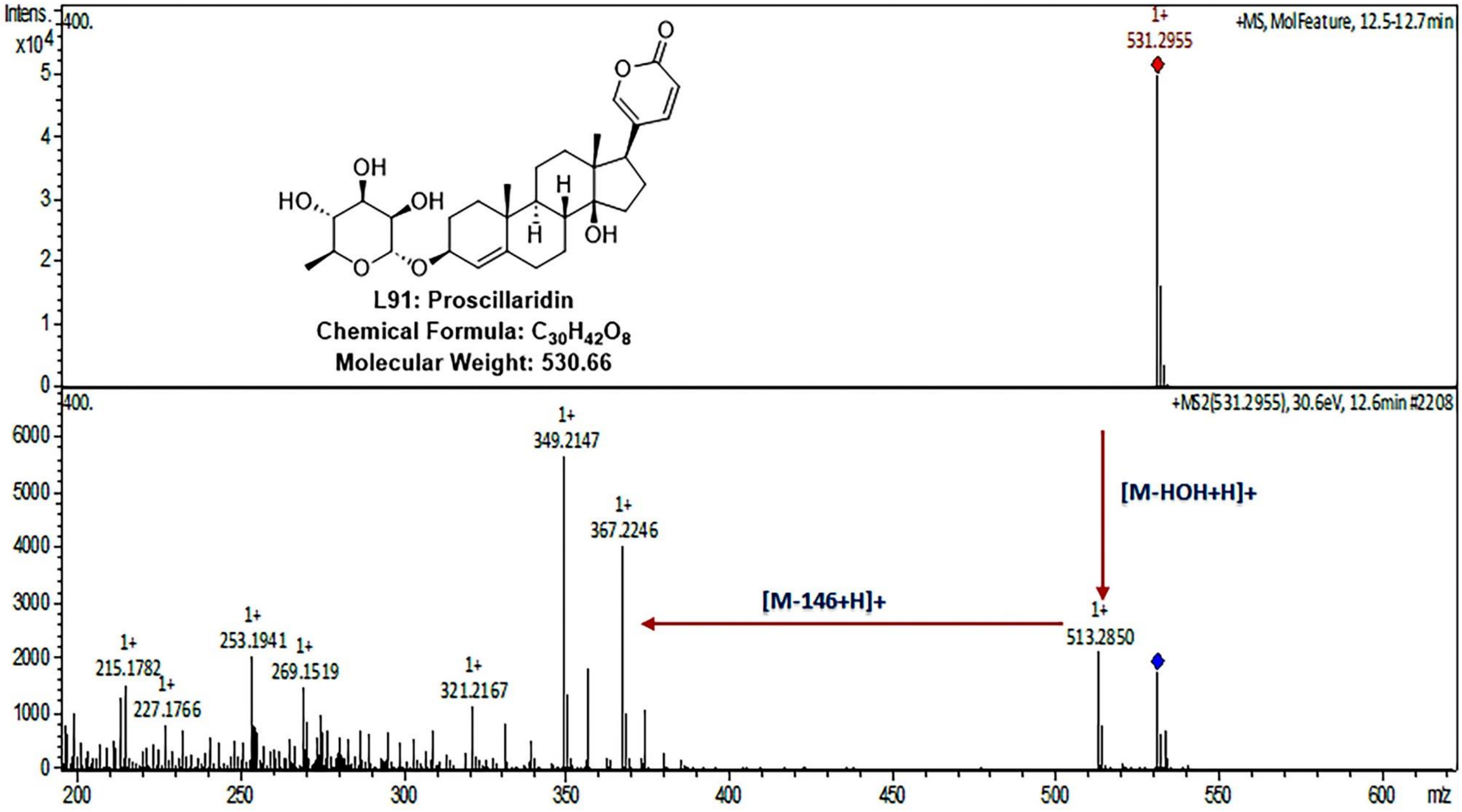

Figure S1.-E: ESI-MS/MS spectrum of L91 in the positive ion mode.

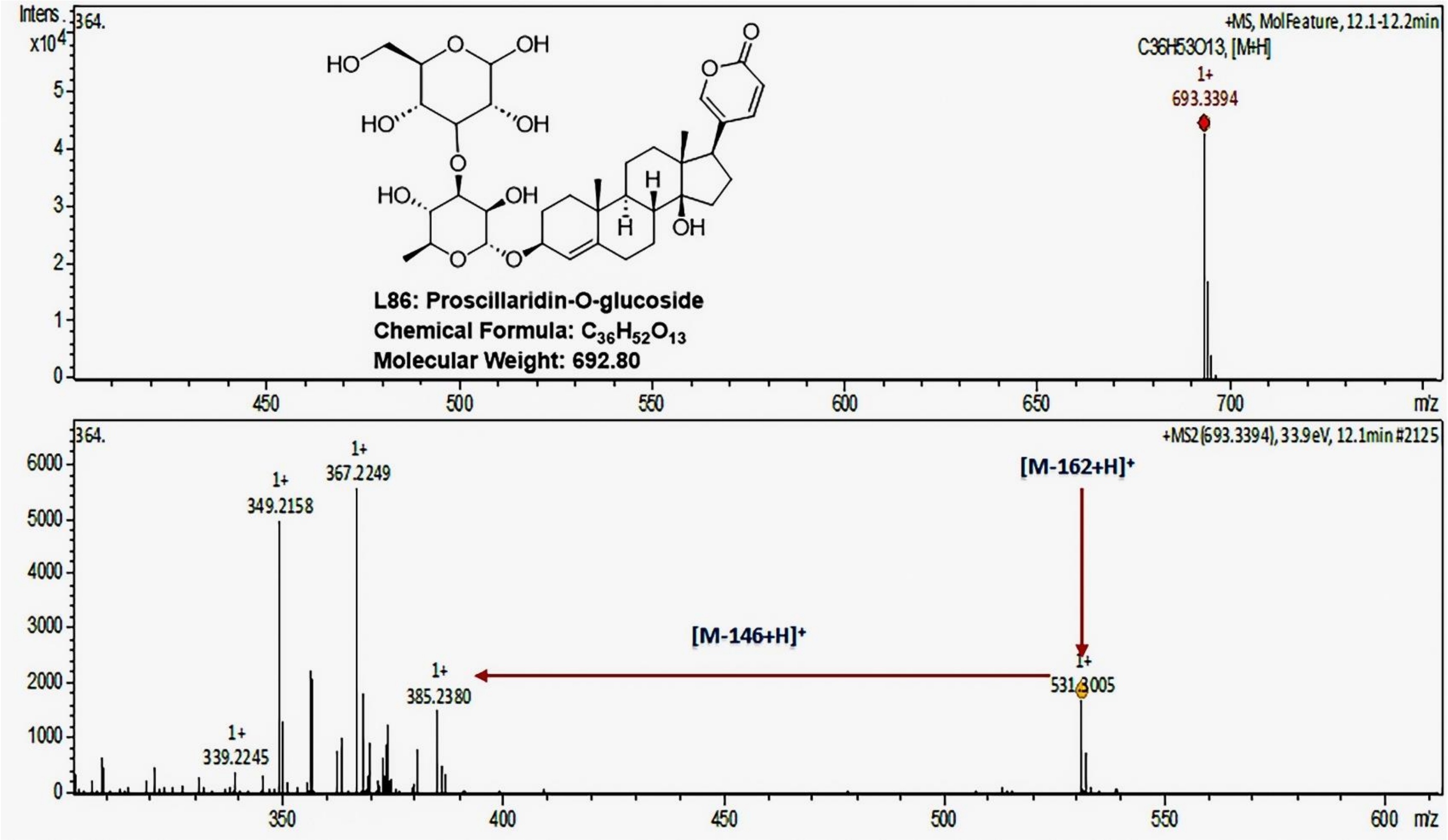

Figure S1.-F: ESI-MS/MS spectrum of L86 in the positive ion mode.

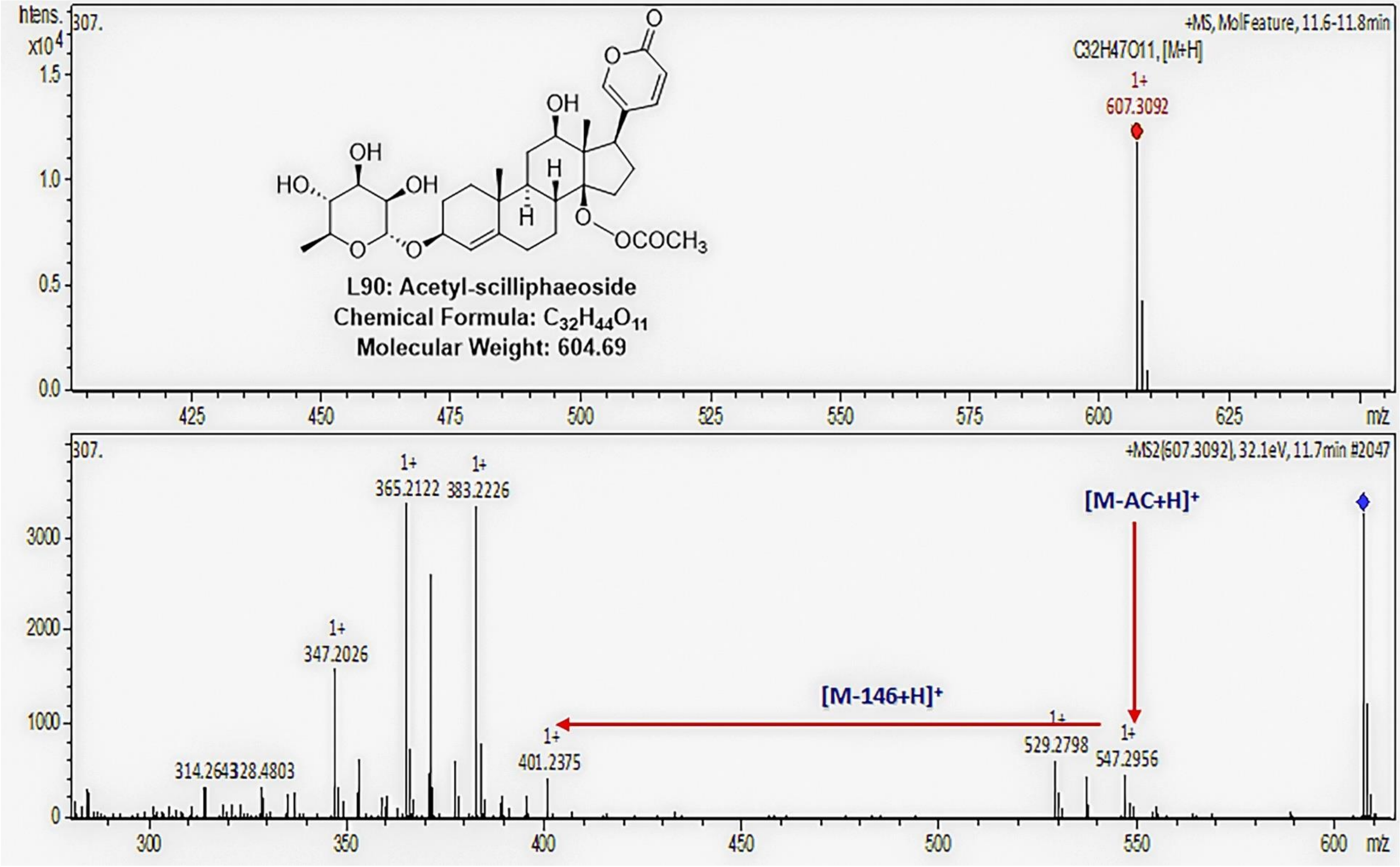

Figure S1.-G: ESI-MS/MS spectrum of L90 in the positive ion mode.

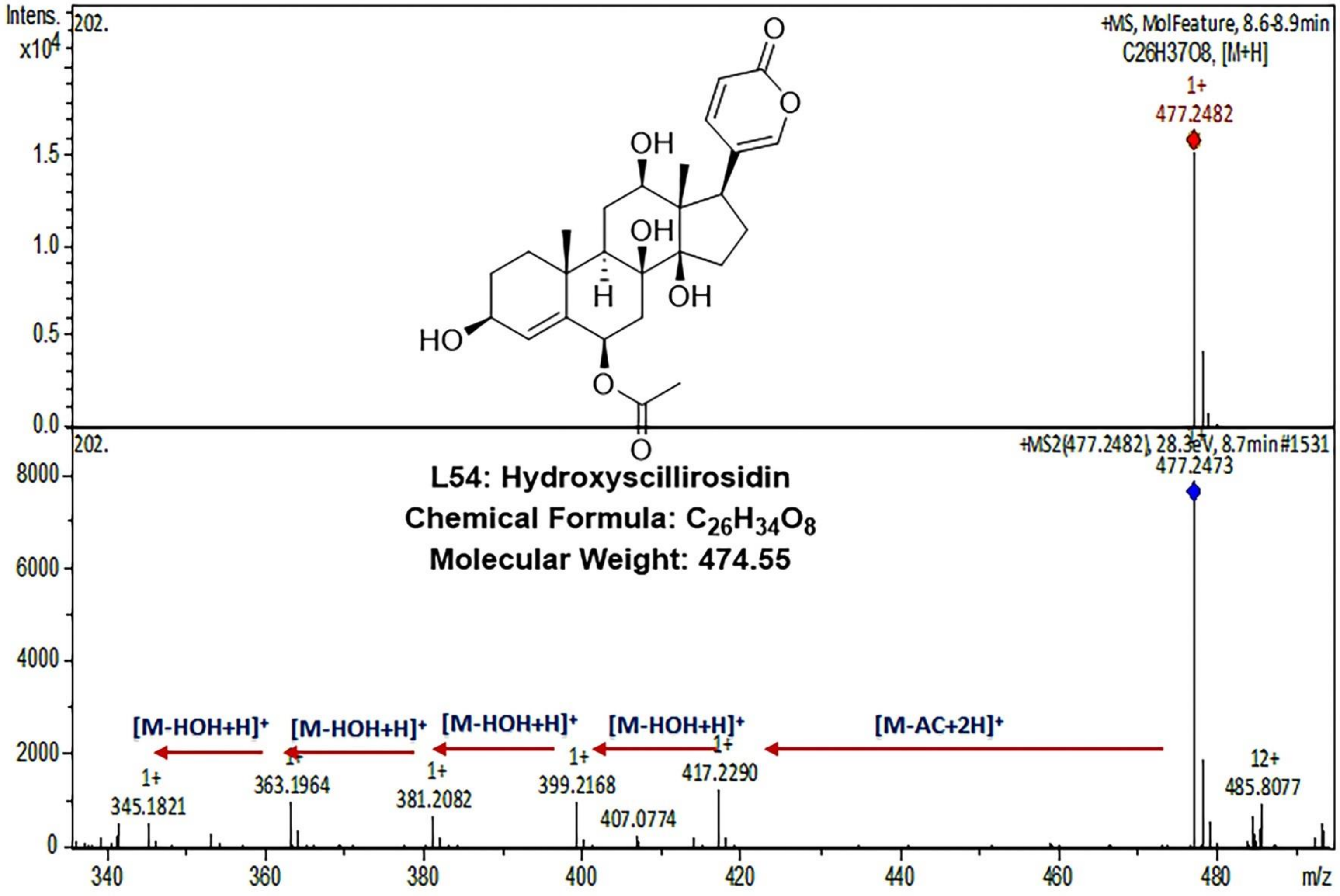

Figure S1.-H: ESI-MS/MS spectrum of L54 in the positive ion mode.

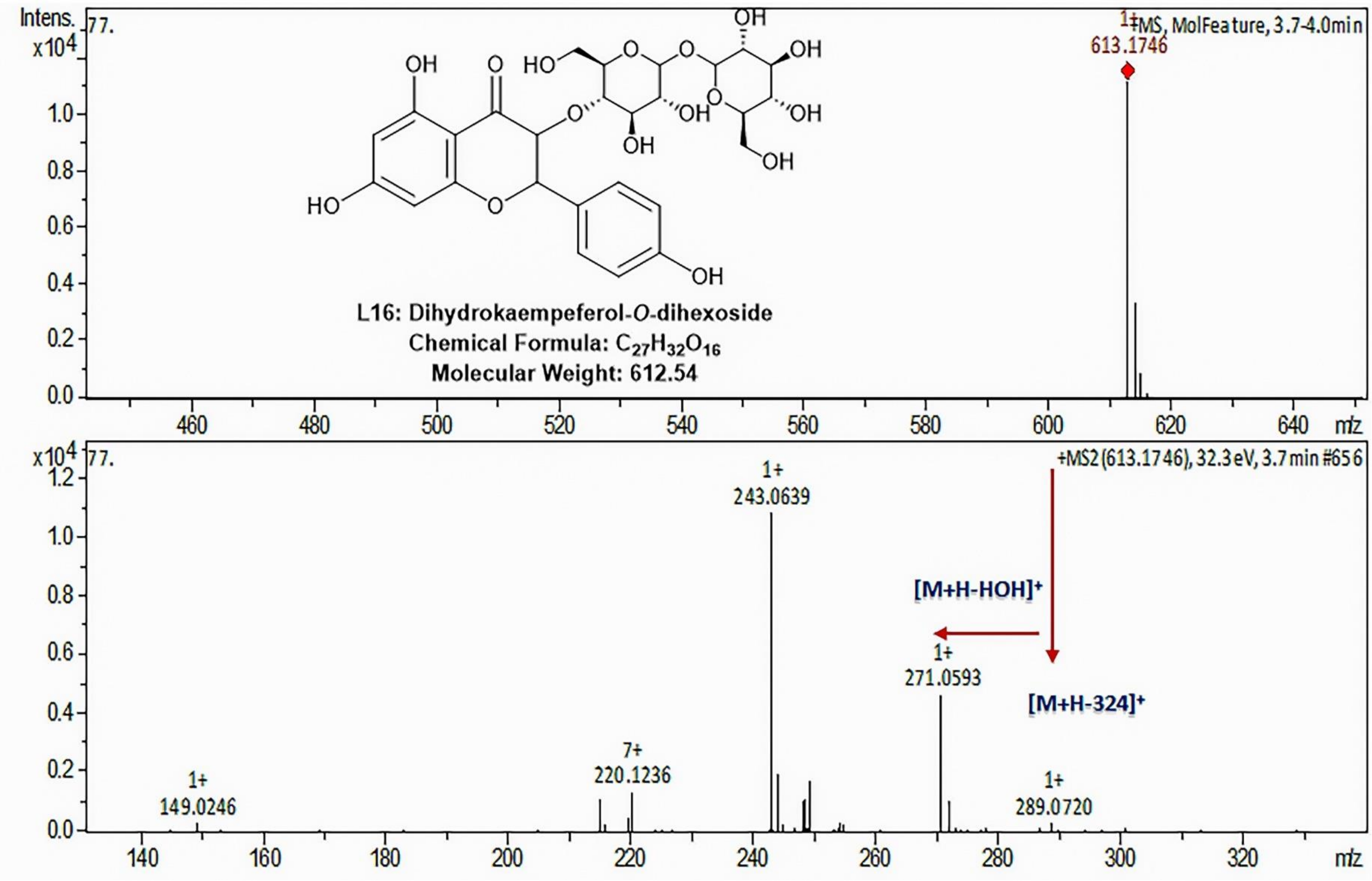

Figure S1.-I: ESI-MS/MS spectrum of L16 in the positive ion mode.

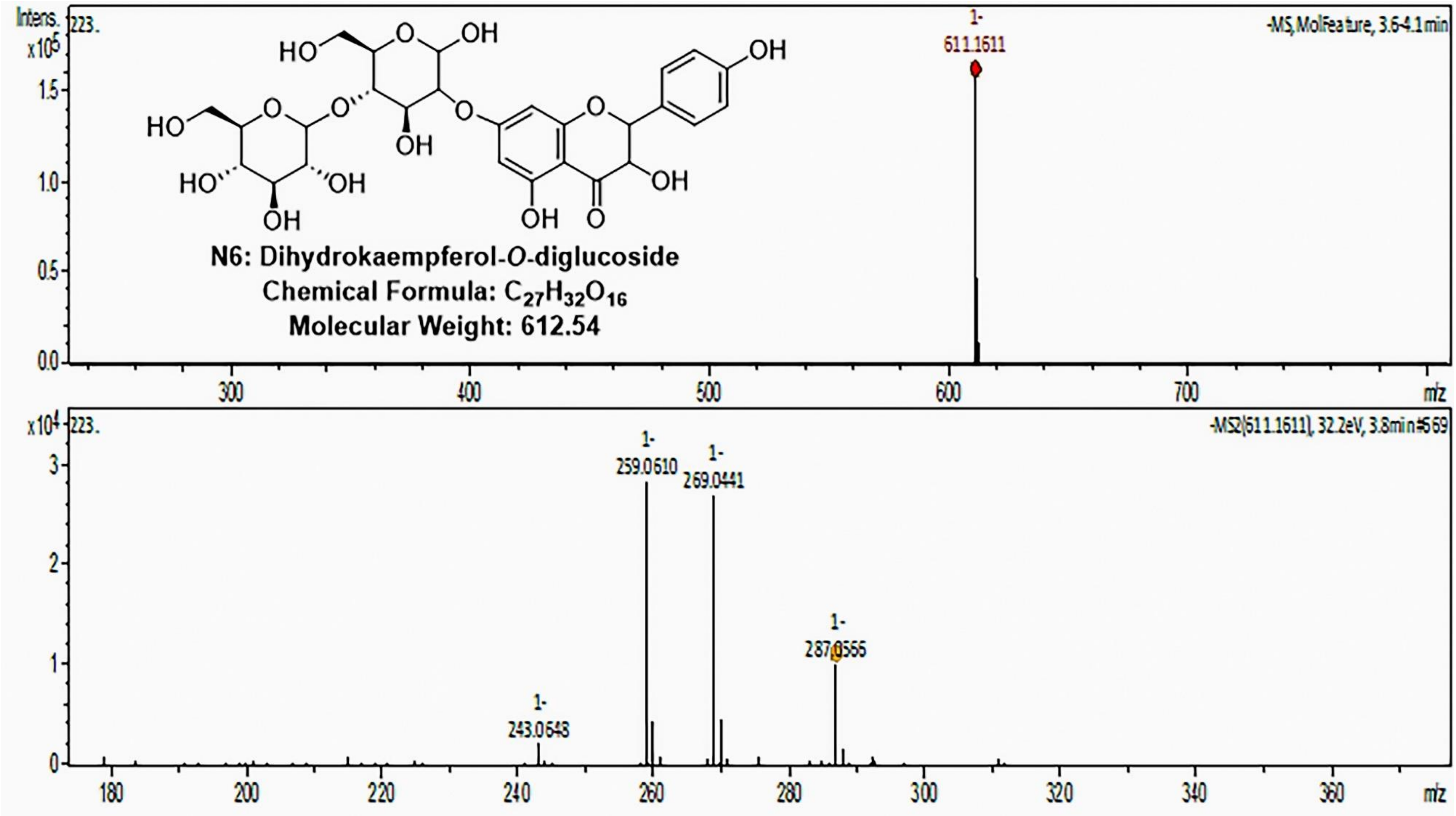

Figure S1.-J: ESI-MS/MS spectrum of N6 in the negative ion mode.

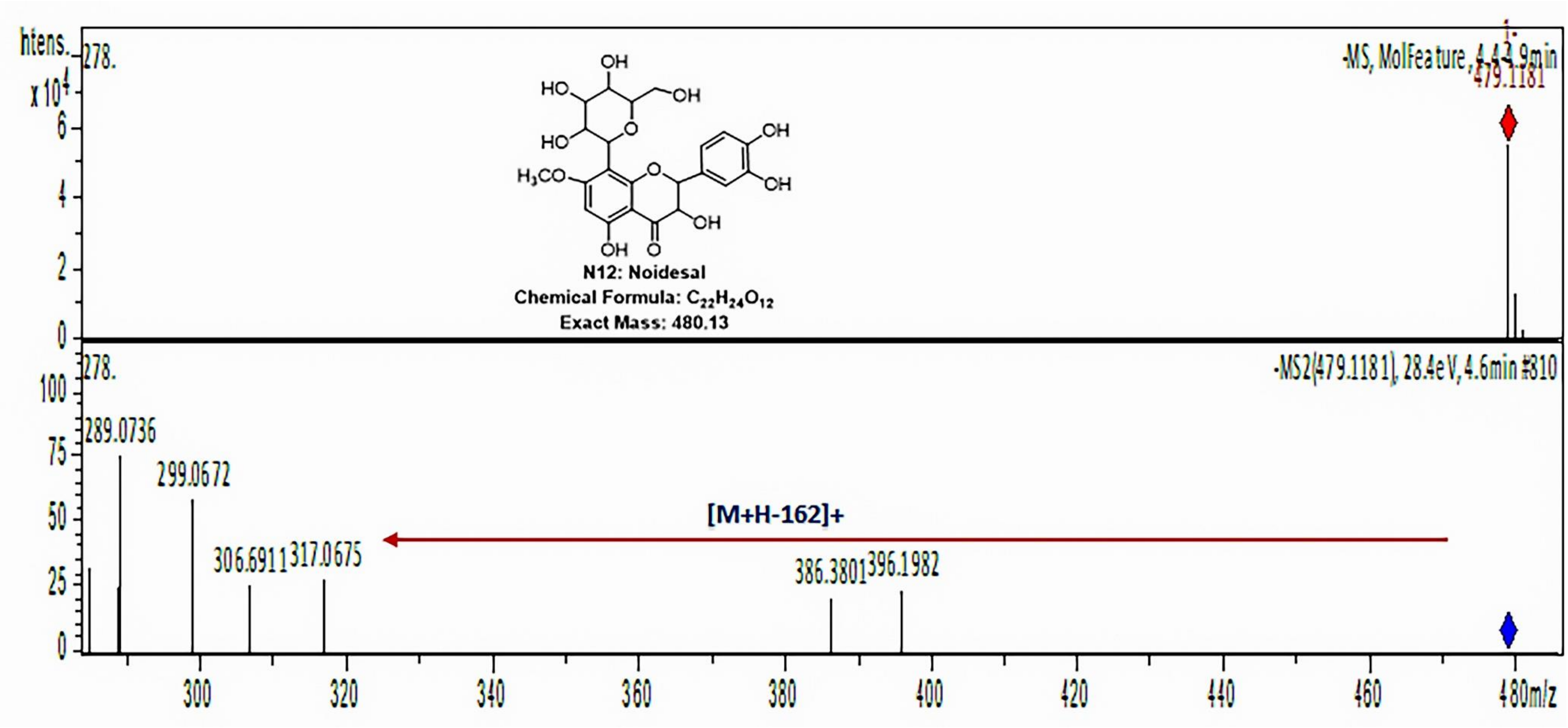

Figure S1.-K: ESI-MS/MS spectrum of N12 in the negative ion mode.

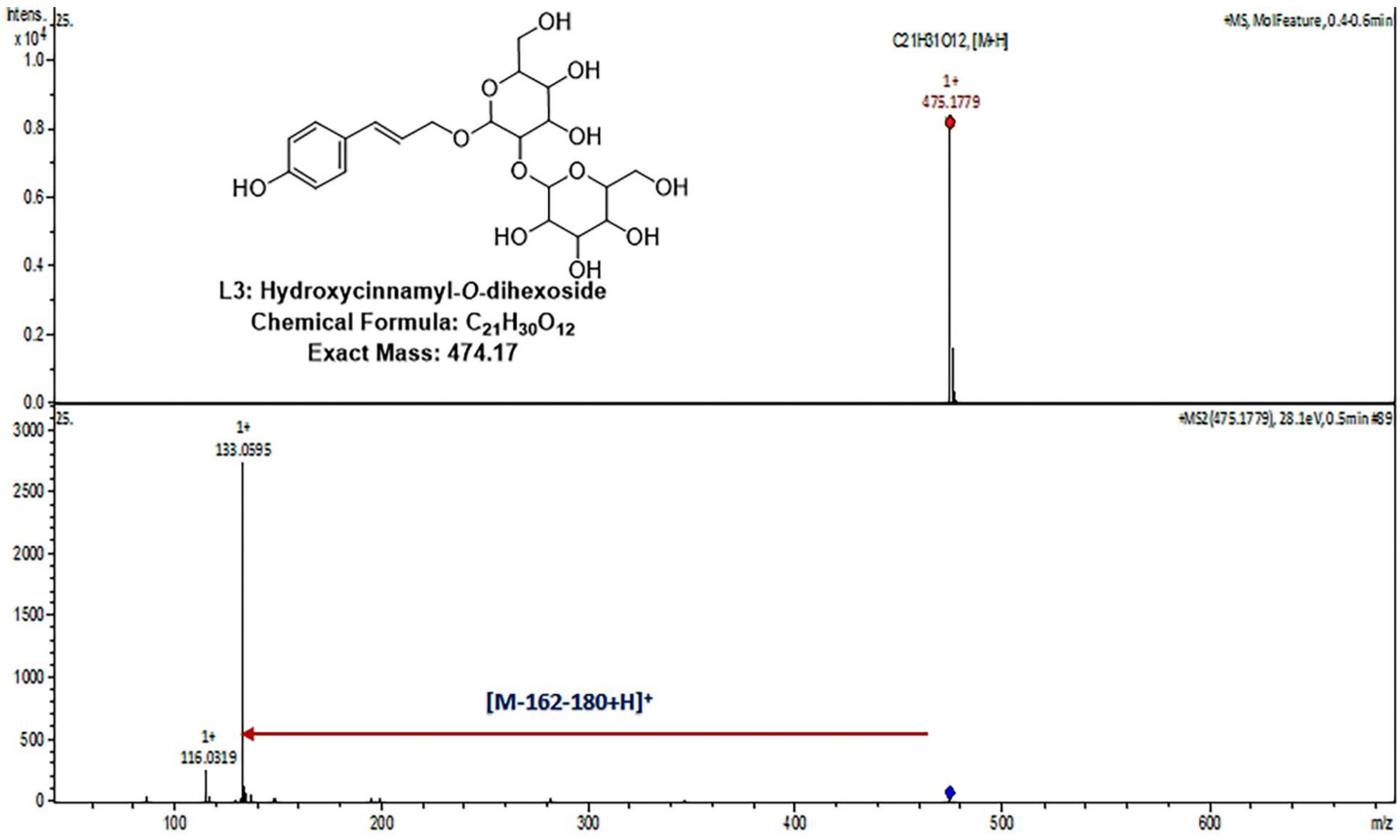

Figure S1.-L: ESI-MS/MS spectrum of L3 in the positive ion mode.

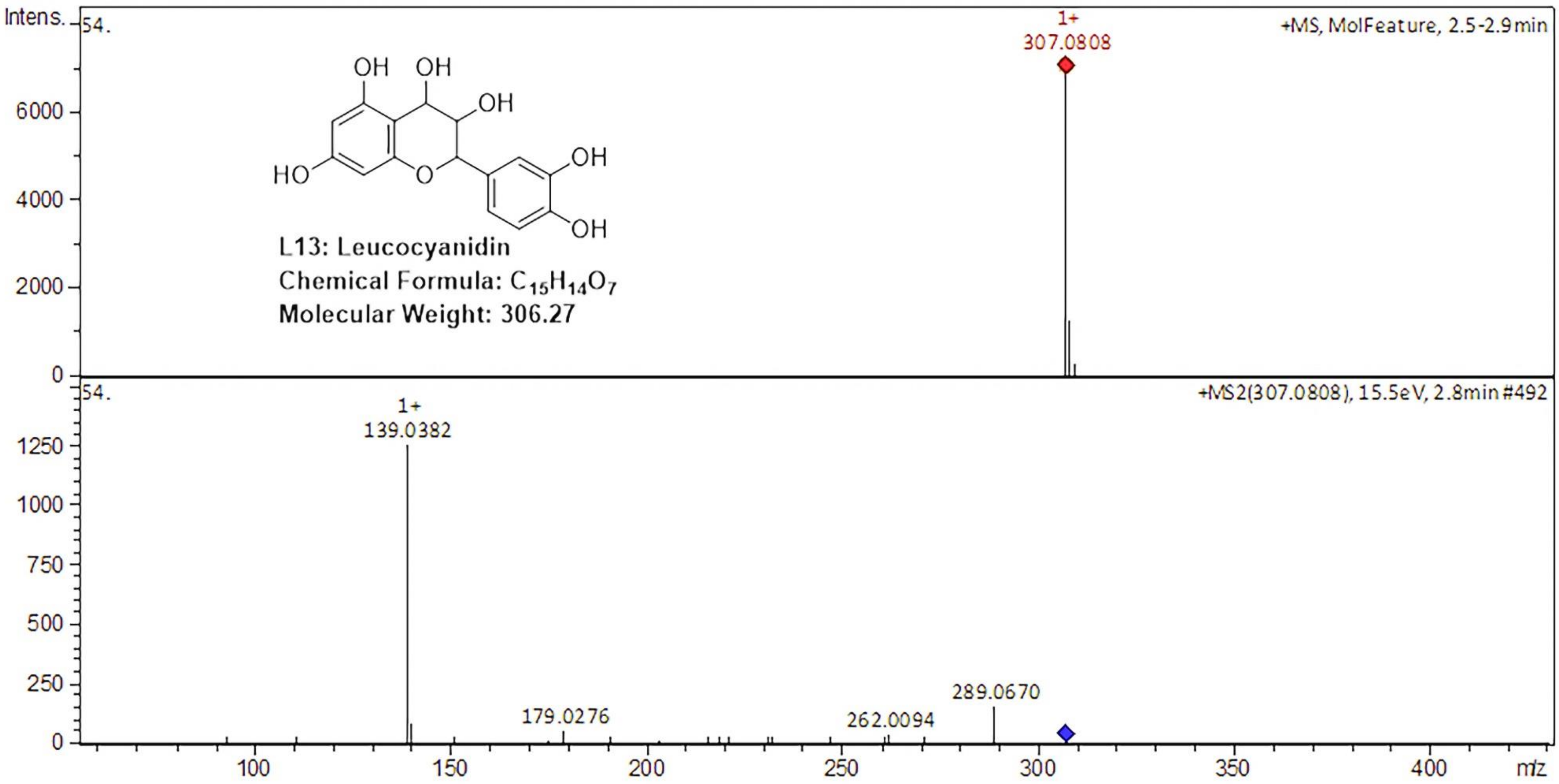

Figure S1.-M: ESI-MS/MS spectrum of L13 in the positive ion mode.

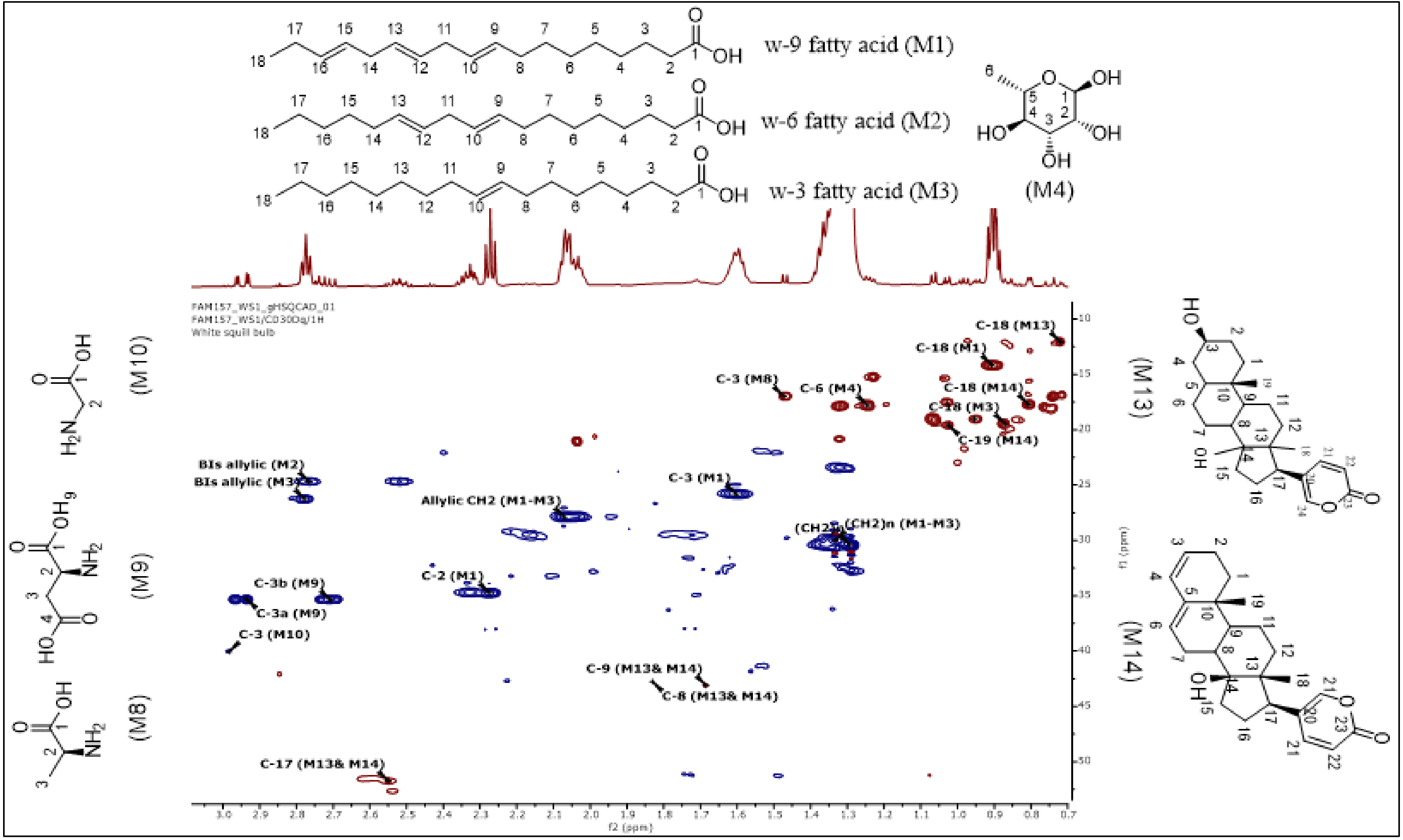

Figure S2. Signal assignment of the ^{1}H-NMR markers for fatty acids (**M1-M3**), rhamnose (**M4**), alanine (**M8**), aspartic acid (**M9**), glycine (**M10**) and bufadienolides (**M13&M14**) using ^{1}H- ^{13}C correlations observed in the HSQC spectrum of squill methanol extract.

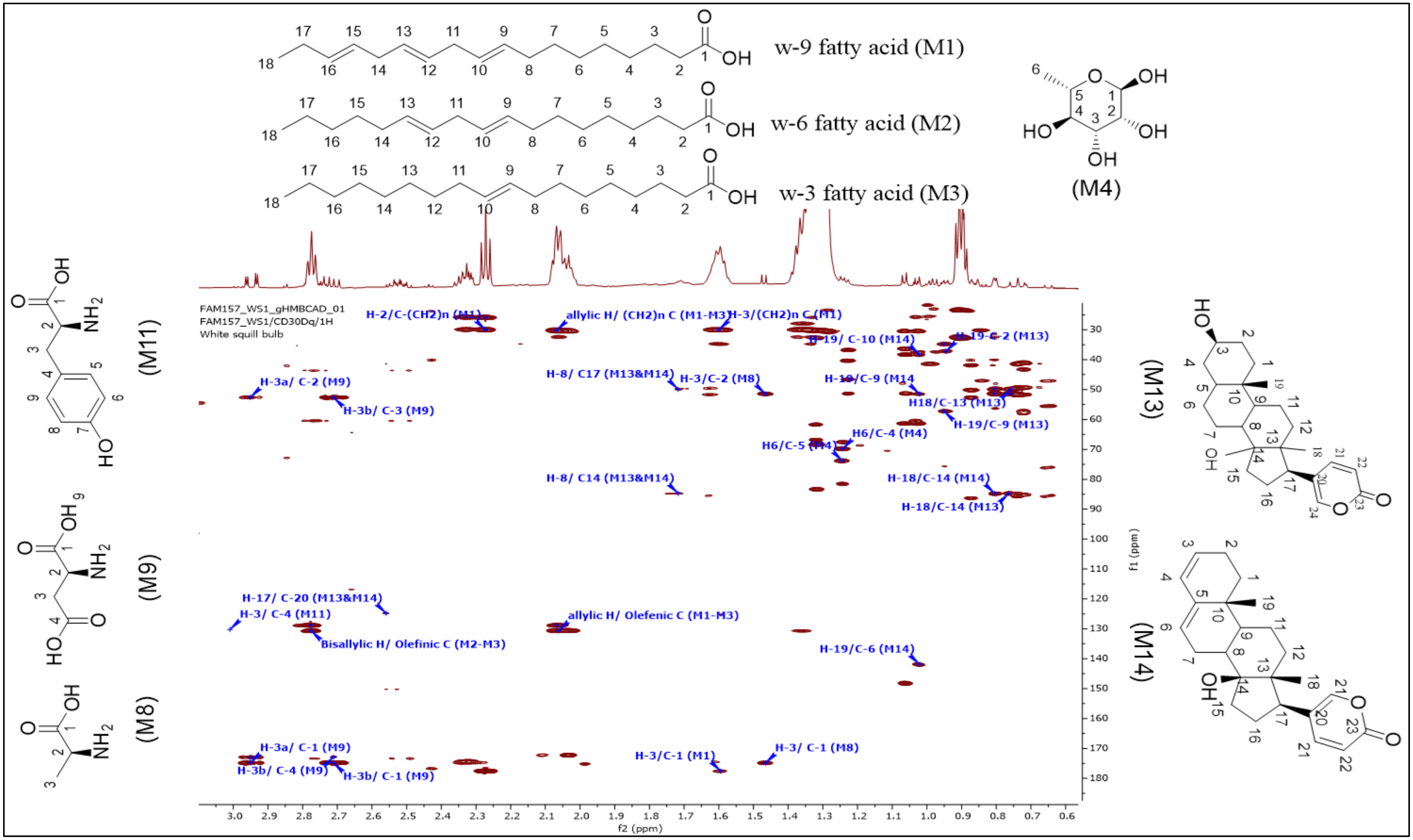

Figure S3. Signal assignment of the ^{1}H-NMR markers for fatty acids (**M1-M3**), rhamnose (**M4**), alanine (**M8**), aspartic acid (**M9**), glycine (**M10**) and bufadienolides (**M13&M14**) using ^{1}H-^{13}C correlations observed in the HMBC spectrum of squill methanol extract.

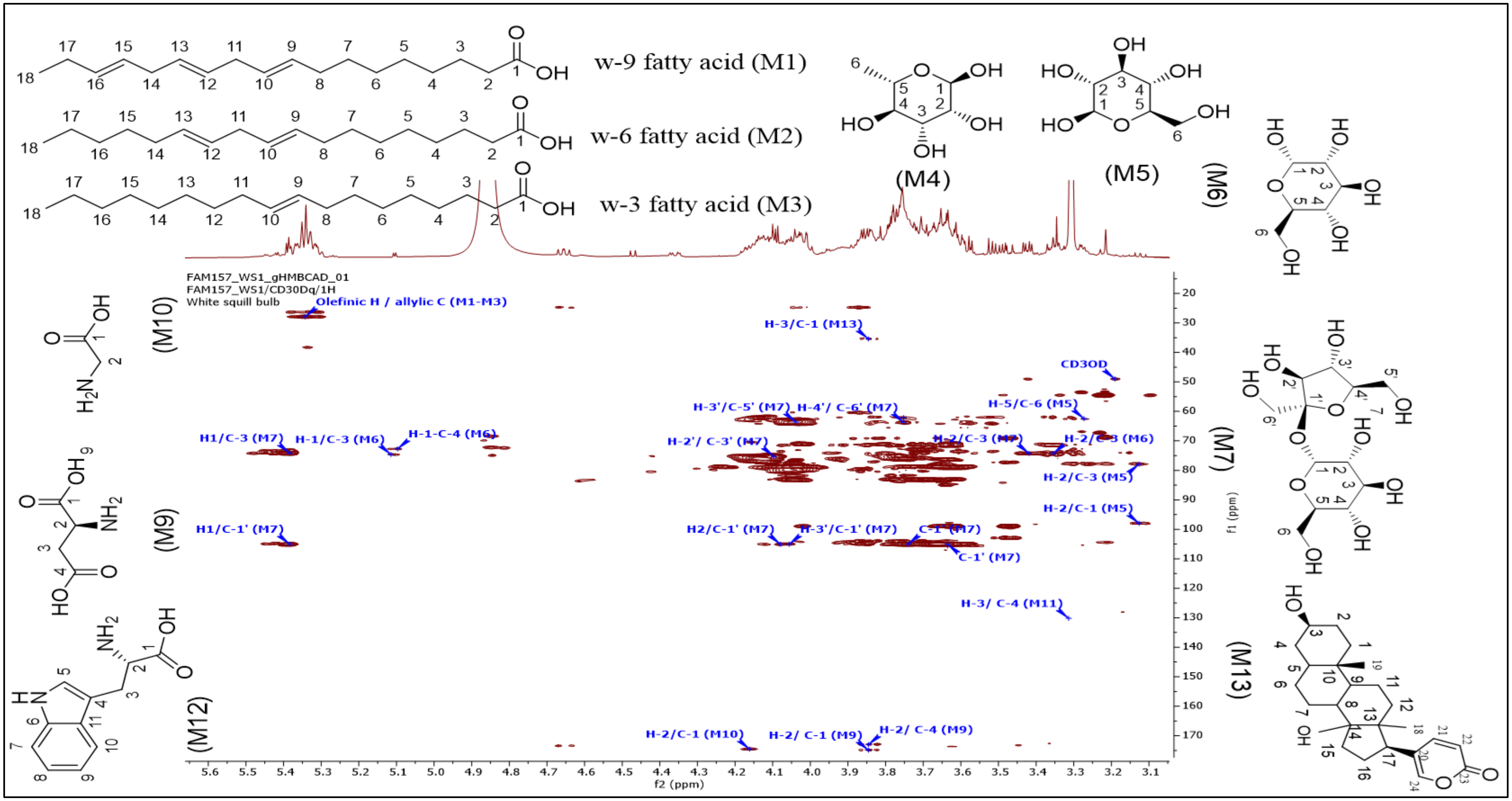

Figure S4. Signal assignment of the ^{1}H-NMR markers for fatty acids (**M1-M3**), rhamnose (**M4**), β, α glucose **(M5& M6)**, sucrose **(M7),** aspartic acid (**M9**), glycine (**M10**), tryptophan **(M12)** and bufalin (**M13**) using ^{1}H-^{13}C correlations observed in the HMBC spectrum of squill methanol extract.

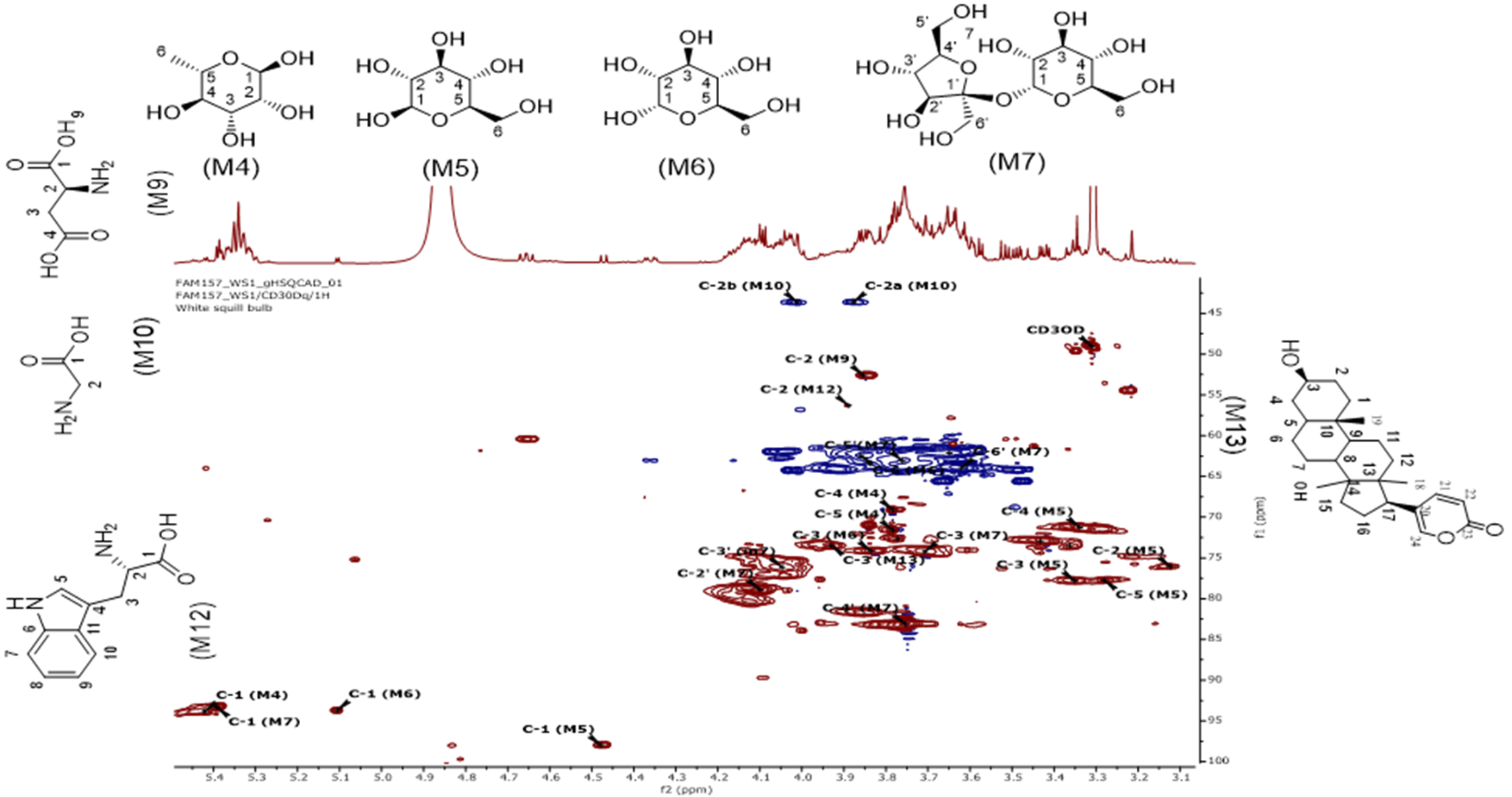

Figure S5. Signal assignment of the ^{1}H-NMR markers for rhamnose (**M4**), β, α glucose **(M5& M6)**, sucrose **(M7),** aspartic acid (**M9**), glycine (**M10**), tryptophan **(M12)** and bufalin (**M13**) using ^{1}H- ^{13}C correlations observed in the HSQC spectrum of squill methanol extract.

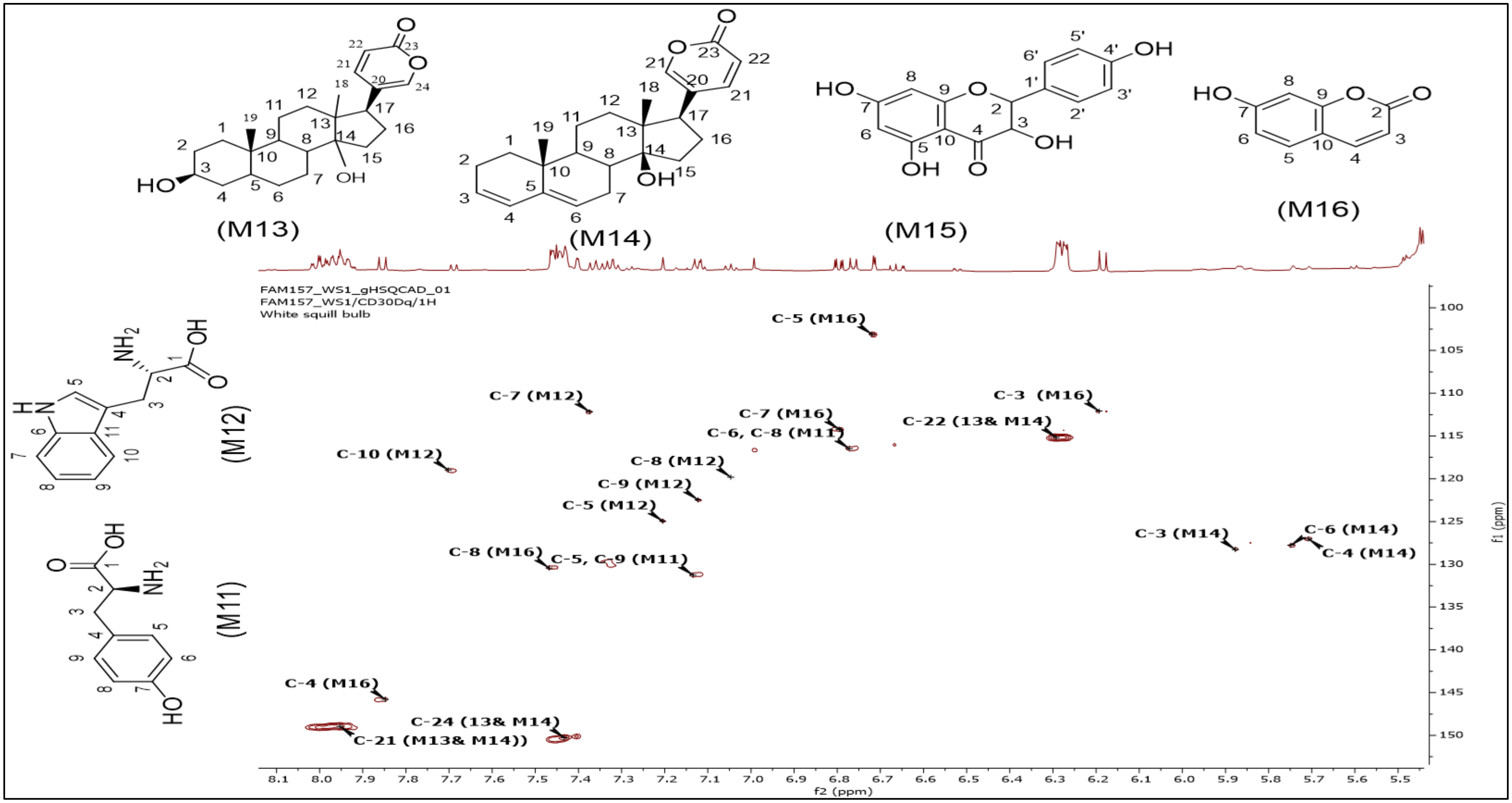

Figure S6: Signal assignment of the ^{1}H-NMR markers for tyrosine (**M11**), tryptophan **(M12)**, bufalin **(M13),** scilliridin (**M14**), dihydro kaempferol (**M15**), and coumarin **(M16)**, ^{1}H-^{13}C correlations observed in the HSQC spectrum of squill methanol extract.

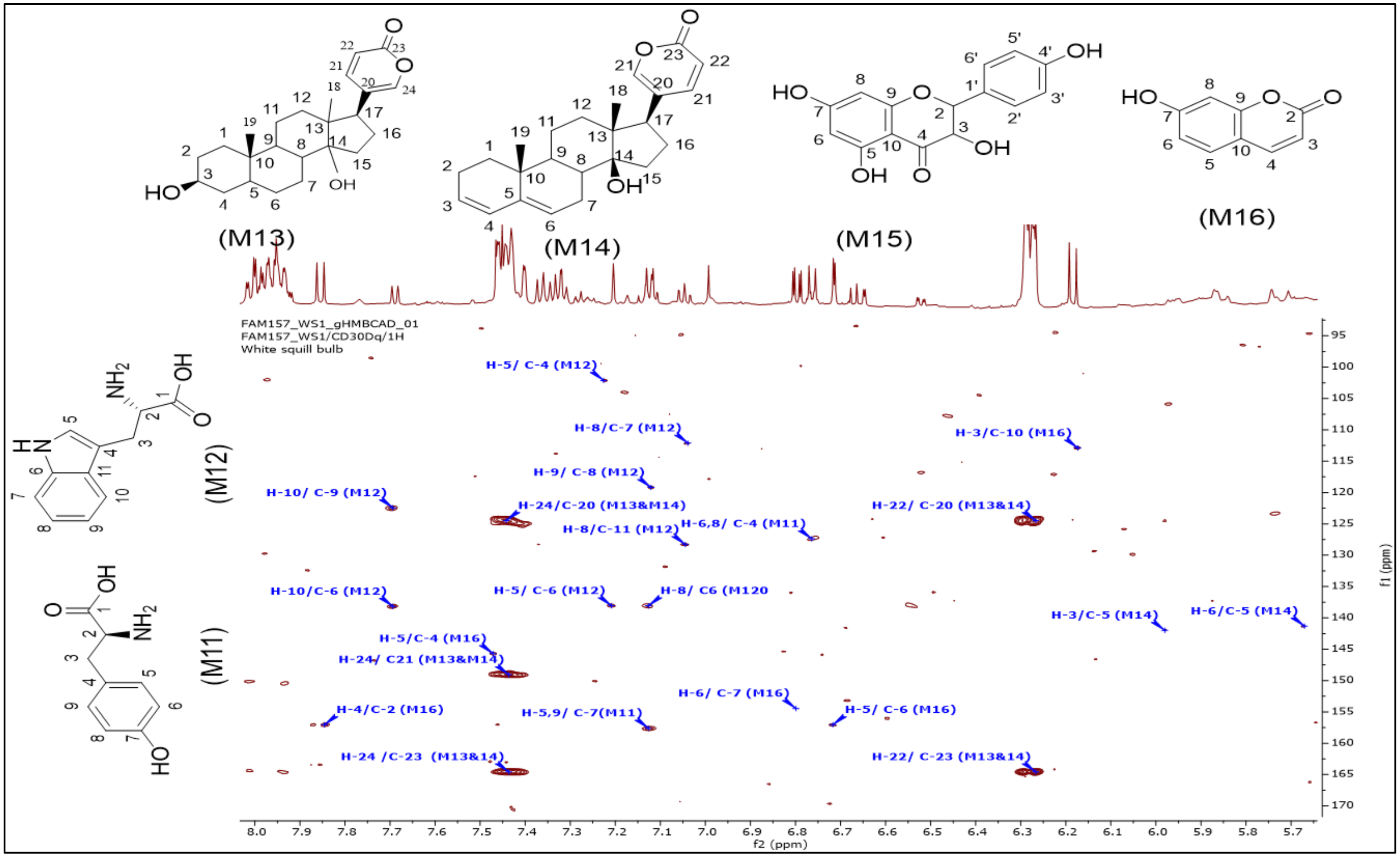

Figure S7. Signal assignment of the ^{1}H-NMR markers for tyrosine (**M11**), tryptophan **(M12)**, bufalin **(M13),** scilliridin (**M14**), dihydro kaempferol (**M15**), and coumarin **(M16)**, ^{1}H-^{13}C correlations observed in the HMBC spectrum of squill methanol extract.

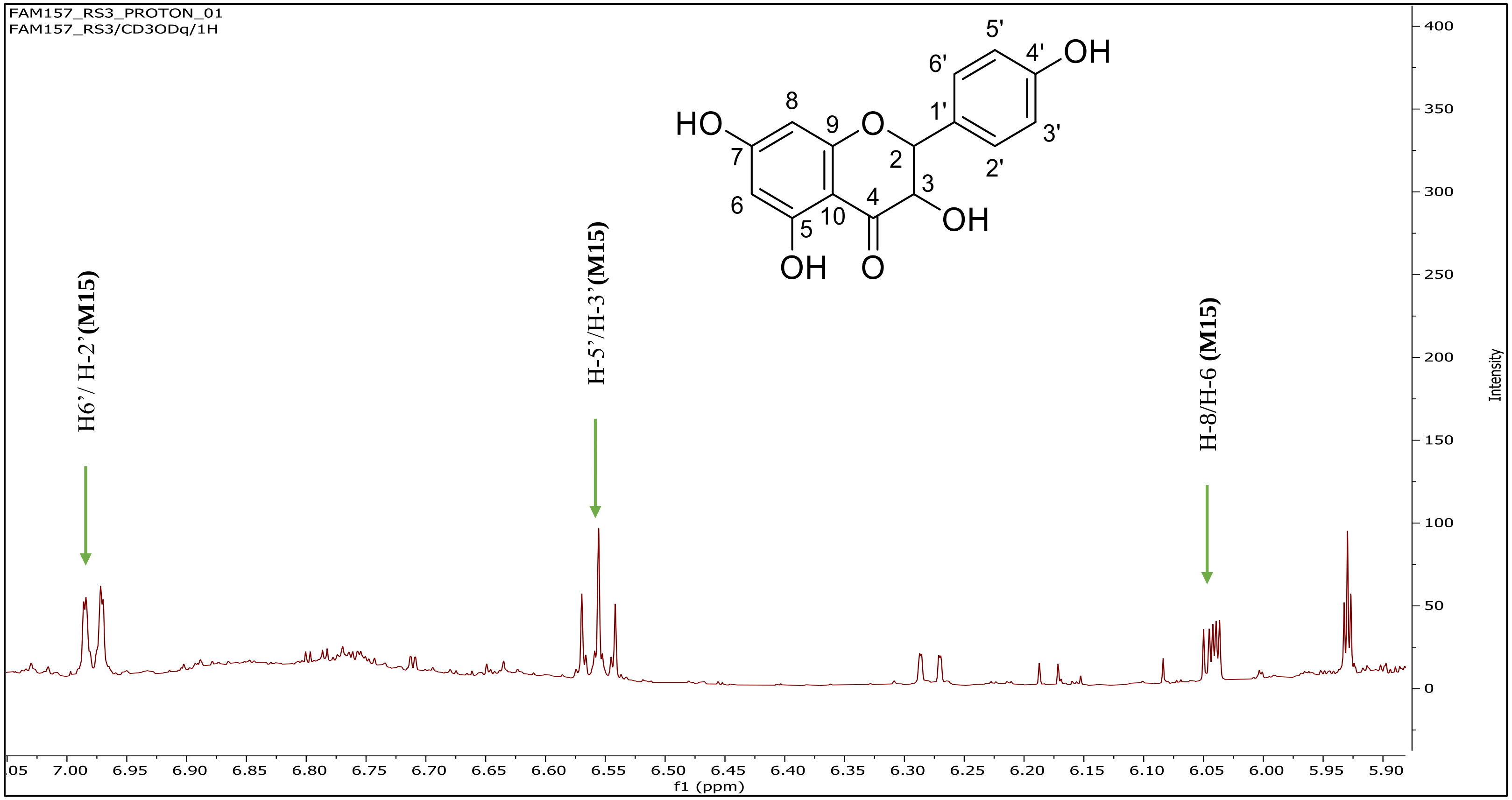

Figure S8. Signal assignment of the proton markers for dihydro kaempferol (**M15**) observed in the ^{1}H-NMR spectrum of RS methanol extract.
